# Distinct evolutionary origins and mixed-mode transmissions of methanogenic endosymbionts are revealed in anaerobic ciliated protists

**DOI:** 10.1007/s42995-025-00295-9

**Published:** 2025-05-13

**Authors:** Tingting Hao, Hua Su, Zijing Quan, Ruixin Zhang, Minjie Yu, Jiahui Xu, Jia Li, Song Li, Alan Warren, Saleh A. Al-Farraj, Zhenzhen Yi

**Affiliations:** 1 Guangzhou Key Laboratory of Subtropical Biodiversity and Biomonitoring, School of Life Science, South China Normal University, Guangzhou, 510631 China; 2 School of Marine Sciences, Sun Yat-Sen University, Zhuhai, 519082 China; 3 Department of Life Sciences, Natural History Museum, London, SW7 5BD UK; 4 Zoology Department, College of Science, King Saud University, 11451 Riyadh, Saudi Arabia

**Keywords:** Anaerobic ciliates, Endosymbiont replacement, Evolution of associations, Methanogenic endosymbionts

## Abstract

Methanogenic endosymbionts are the only known intracellular archaeans and are especially common in anaerobic ciliated protists. Studies on the evolution of associations between anaerobic ciliates and their methanogenic endosymbionts offer an excellent opportunity to broaden our knowledge about symbiosis theory and adaptation of eukaryotes to anoxic environments. Here, the diversity of methanogenic endosymbionts was analyzed with the addition of nine anaerobic ciliate populations that were newly studied by various methods. Results showed that diverse anaerobic ciliates host methanogenic endosymbionts that are limited to a few genera in orders Methanomicrobiales, Methanobacteriales, and Methanosarcinales. For the first time, anaerobic ciliates of the classes Muranotrichea and Prostomatea were found to host methanogenic endosymbionts. Distinct origins of endosymbiosis were revealed for classes Armophorea and Plagiopylea. We posit that armophoreans and plagiopyleans might have harbored *Methanoregula* (order Methanomicrobiales) and *Methanocorpusculum* (order Methanomicrobiales), respectively, as methanogenic endosymbionts at the beginning of their evolution. Subsequently, independent endosymbiont replacement events occurred in methanogen-ciliate associations, probably due to ecological transitions, species radiation of ciliate hosts, and vertical transmission bottlenecks of endosymbionts. Our results shed light on the evolution of associations between anaerobic ciliates and methanogens, and identifies the necessary preconditions for illustrating mechanisms by which endosymbioses between these partners were established.

**Supplementary Information:**

The online version contains supplementary material available at 10.1007/s42995-025-00295-9.

## Introduction

Symbiotic events play vital roles in ecology and evolution, including facilitating hosts to occupy new ecological niches and promoting cellular evolution in eukaryotes (Dziallas et al. 2012; Husnik et al. 2021; Kim et al. 2024; Nowack and Melkonian 2010). Unicellular eukaryotes (protists) are suitable hosts for prokaryotic endosymbionts given: (1) their habitat almost invariably contains abundant prokaryotes; and (2) their ability to phagocytose particulate matter such as prokaryotic cells (Dziallas et al. 2012; Tang et al. 2023). Associations with endosymbiotic prokaryotes are widely spread across the protist tree of life (Husnik et al. 2021). Hence, studies focusing on the diversity of protist symbioses are necessary to broaden the theory of endosymbiotic transmission.

Methanogenic endosymbionts of protists are the only known examples of intracellular archaeans (Beinart et al. 2018). It is generally accepted that certain methanogens ingested by particular phagotrophic protists might occasionally escape digestion within food vacuoles, survive in the cytoplasm, and go on to form stable symbiotic relationships with their protist hosts (Dziallas et al. 2012). Methanogenic endosymbionts help reduce the partial pressure of hydrogen, which is beneficial for the functioning of mitochondrion-related organelles (MROs) that produce H_2_ gas in anaerobic protists (van Hoek et al. 2000). Accordingly, methanogenic endosymbionts facilitate adaptation by protists to anoxic environments. Methanogenic endosymbionts have been found in a diverse array of anaerobic protists, and are especially common in anaerobic ciliates (Fenchel and Finlay 2010). Previous studies demonstrated that the growth rate of some anaerobic ciliates is much lower if their methanogenic endosymbionts are inhibited (Fenchel and Finlay 1991, 1992).

Numerous studies have reported that anaerobic ciliates host methanogenic endosymbionts (e.g., Clarke et al. 1993; Embley and Finlay 1994; Hackstein 2018; Lewis et al. 2018; Rotterová et al. 2020; van Hoek et al. 2000; Zhuang et al. 2022). Nevertheless, our understanding of the evolution of associations between anaerobic ciliates and their methanogenic endosymbionts remains limited (Hirakata et al. 2015). Among the 14 ciliate classes that have anaerobic species, at least six (viz. Armophorea, Karyorelictea, Oligohymenophorea, Parablepharismea, Litostomatea, and Plagiopylea) include examples that host methanogenic endosymbionts in their cytoplasm (Supplementary Table S2; Beinart et al. 2018; Edgcomb et al. 2011; Embley et al. 1992a, b, Embley and Finlay 1994; Finlay et al. 1993; Gijzen et al. 1991; Hirakata et al. 2015; Lewis et al. 2018; Lind et al. 2018; Méndez-Sánchez et al. 2024; Narayanan et al. 2009; Nitla et al. 2019; Schrecengost et al. 2024; Shinzato et al. 2007; Takeshita et al. 2019; van Bruggen et al. 1984, 1986; van Hoek et al. 2000). Methanogenic endosymbionts and MROs that produce H_2_ gas are often simultaneously present in ciliates, and the presence of both is crucial to ciliate adaptation to anoxia (Rotterová et al. 2022). Recent studies have revealed that transitions from mitochondria to MROs within ciliates are group-specific and may have occurred multiple times (Chen et al. 2022; Rotterová et al. 2022). Hence, it is likely that ancestors of different ciliate classes independently acquired methanogenic endosymbionts, considering that a low intracellular hydrogen partial pressure is necessary for the functioning of MROs that produce H_2_ gas. Consequently, investigations that include at least two distantly related ciliate classes are needed to test this hypothesis. Early studies have suggested that the ancestor of anaerobic armophorean ciliates hosted methanogenic endosymbionts, and then multiple acquisitions and replacements occurred during the adaptation of these ciliates to habitats such as freshwater sediments and the intestinal tracts of animals (Hackstein 2018; van Hoek et al. 2000). Fortunately, dense taxon sampling of the phylogenetically distant anaerobic ciliate classes Armophorea and Plagiopylea as well as their corresponding methanogenic endosymbionts were performed in recent investigations, significantly improving our understanding about the evolution of associations between these symbiotic partners (Méndez-Sánchez et al. 2024; Schrecengost et al. 2024). It has been revealed that most armophoreans isolated from freshwater/soil usually host *Methanobacterium* or *Methanoregula*, and armophoreans isolated from marine/brackish water, and most plagiopyleans isolated from marine/brackish water, specifically host *Methanocorpusculum* (Méndez-Sánchez et al. 2024; Schrecengost et al. 2024). Furthermore, these studies also demonstrated mixed-mode transitions, and transitions that are habitat specific and host specific at taxonomic ranks below that of class (Méndez-Sánchez et al. 2024; Schrecengost et al. 2024).

In this study, identifications of methanogenic endosymbionts from three armophorean species, four populations of three plagiopylean species, one prostomatean species, and one muranotrichean species were made for the first time using autofluorescence, fluorescence in situ hybridization (FISH), amplicon sequencing (~ 400 bp), and 16S rDNA sequences (~ 1 kb). Subsequently, analyses of the evolutionary history of endosymbiotic associations between these host ciliates and their methanogenic endosymbionts were performed. Our study mainly focuses on general patterns of the evolution of associations between anaerobic ciliates and their methanogenic endosymbionts at high taxonomic rank (class), with emphasis on comparing evolutionary events between the classes Armophorea and Plagiopylea. The main aim is to broaden our knowledge of the associations between anaerobic ciliates and their methanogenic endosymbionts.

## Materials and methods

### Ciliate sampling and culture

Nine anaerobic ciliate populations were collected from coastal locations in China during the period February 2019 to March 2021 (Supplementary Table S1). For each sample, both mud/sediment and water were collected, and the sampling bottles were completely filled. The dissolved oxygen concentration (DO) and salinity of water at each sampling site were measured with an ORION 520 M-01A (Thermo Fisher Scientific, MA, USA). *Metopus laminarius* was cultured in CMV medium (Narayanan et al. 2007), whereas the other eight populations were cultured in artificial seawater (Su et al. 2024). In both cases, the media were flushed with nitrogen gas to ensure anaerobic conditions and then transferred into cell culture flasks at room temperature (ca. 25 ℃) (Chen et al. 2022). For all species, rice grains were added to enrich bacteria as food for ciliates.

### Observations using light microscopy, epifluorescence microscopy, and transmission electron microscopy (TEM)

**Fig. 1:**
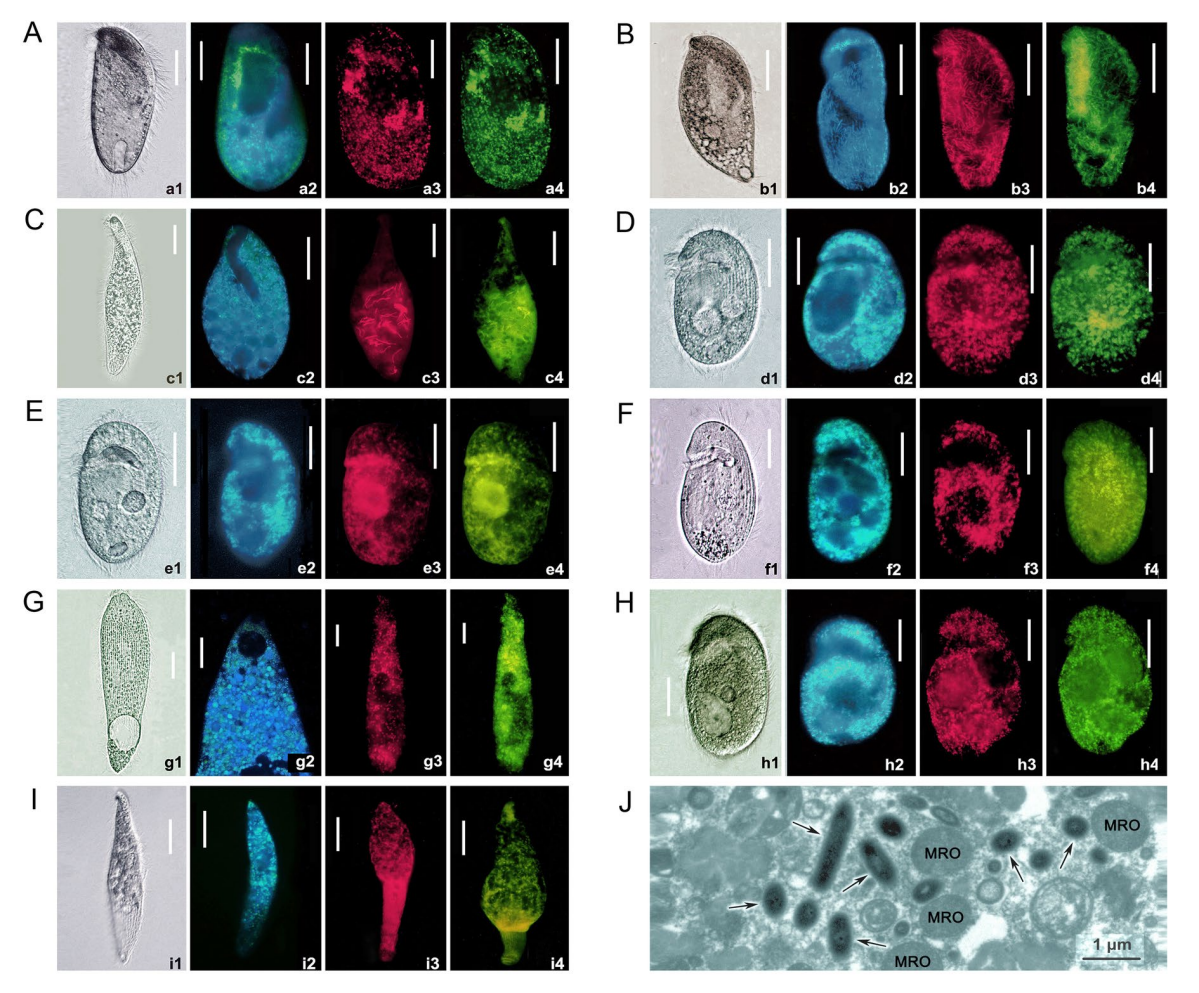
Photomicrographs of free-living anaerobic ciliates from life (a1, b1, c1, d1, e1, f1, g1, h1, i1), autofluorescence (a2, b2, c2, d2, e2, f2, g2, h2, i2), and fluorescence in situ hybridization (FISH) (a3–4, b3–4, c3–4, d3–4, e3–4, f3–4, g3–4, h3–4, i3–4). *Metopus* sp. (**A**), *M.* cf. *contortus* (**B**), *M. laminarius* (**C**), *Plagiopyla* cf. *narasimhamurtii* pop. 1 (**D**), *P.* cf. *narasimhamurtii* pop. 2 (**E**), *P.* cf. *ramani* (**F**), *Apolagynus* cf. *cucumis* (**G**), *P. marina* (**H**), *Thigmothrix strigosa* (**I**). Archaeal probe ARCH915 (a3, b3, c3, d3, e3, f3, g3, h3, i3), Methanomicrobiales-specific probe MG1200b (a4, d4, e4, h4, f4, g4, i4), Methanobacteriales-specific probe MB311 (b4, c4). TEM photomicrographs of *M.* cf. *contortus* (**J**). Black arrows, endosymbionts. MRO, mitochondrion-related organelles. Scale bars: 20 μm (a1–4, b1–4, c1–4, d1–4, e1–4, f1–4, g1, 3–4, h1–4); 1 μm (**J**)

For each species, about 10 cells were starved for 5 h and then washed three times with their sterile culture medium. Live ciliates were observed using a bright-field microscopy. The autofluorescence signal for F420 coenzyme of methanogenic endosymbionts was detected in living ciliate cells using fluorescence microscopy (Nikon 80i) with a UV filter (Doddema and Vogels 1978). As described in previous investigations (Jiang et al. 2023; Jin et al. 2023; Lu et al. 2023), ciliate species identification was based on 18S rDNA sequences and morphological characters observed in vivo and/or after silver staining (Fig. 1; Supplementary Fig. S1). Among the nine ciliate populations, *Metopus laminarius*, *Plagiopyla* cf. *narasimhamurtii* pop. 2, and *Apolagynus* cf. *cucumis* had been identified and reported in previous investigations (Chen et al. 2022; Xu et al. 2025). Approximately 50,000 cells of *M.* cf. *contortus* were centrifuged at 4500 *g* for 7 min and transferred into a 1.5 mL EP tube as described previously (Su et al. 2023). Glutaraldehyde at a concentration of 2.5% was added to fix the cells for 2 h. Ultra-thin sections were cut and observed using a Hitachi HT-7800 transmission electron microscope at an accelerating voltage of 80 kV at Wuhan Servicebio Technology Co., Ltd.

### DNA extraction, PCR amplification, and sequencing

To minimize contamination by prokaryotes, target ciliates were first transferred to clean medium without food using glass micropipettes and then washed at least five times. Genomic DNA of ciliates was extracted using a REDExtract- *N*-Amp^TM^Tissue PCR Kit (SIGMA, USA) following the manufacturer’s protocol. For DNA extraction of free-living bacteria and archaea in samples from each sampling site, the process was as follows: the sample bottles were kept upright for 30 min allowing sediment and ciliates to precipitate by gravity; the supernatant was collected, and the few ciliates remaining in the supernatant were quickly removed under a stereomicroscope using a micropipette; the supernatant without ciliates was centrifuged at 4800 *g* for 2 min at room temperature; the DNA of free-living bacteria and archaea was extracted using the same kit as mentioned above.

The 16S rDNA V4 regions (~ 400 bp) both of intracellular archaea within ciliates and of free-living archaea in corresponding ciliate-free culture media were amplified using universal archaeal primers (Parch519f: 5ʹ-CAGCCGCCGCGGTAA-3ʹ; ARC915r: 5ʹ-GTGCTCCCCCGCCAATTCCT-3ʹ) (Coolen et al. 2004; Øvreås et al. 1997). The PCR reaction was performed using Q5 High-Fidelity DNA Polymerase (New England BioLabs, USA) with the following protocol: initial denaturation at 98 °C for 30 s, 35 cycles of denaturation at 98° C for 15 s, annealing at 67 °C for 30 s, extension at 72 °C for 1 min, and a final extension at 72 °C for 10 min. Amplified PCR products were detected by 1.00% agarose gel electrophoresis and sequenced on an Illumina Novaseq 6000 PE250 platform (Guangdong Magigene Biotechnology Co. Ltd., China).

The ciliate 18S rDNA was amplified using the universal eukaryotic primers (82F: 5ʹ-GAAACTGCGAATGGCTC-3ʹ; 18SR: 5ʹ-AACCTGGTTGATCCTGCCAGT-3’) (López-García et al. 2003; Medlin et al. 1988). The PCR reaction was performed as described in Chen et al. (2022). The 18S rDNA sequences of six ciliates, namely *Metopus* sp., *M.* cf. *contortus*, *P. marina*, *P.* cf. *narasimhamurtii* pop. 1, *P.* cf. *ramani* and *Thigmothrix strigosa*, were newly obtained in this study. The 18S rDNA sequences of *M. laminarius*, *P.* cf. *narasimhamurtii* pop. 2, and *Apolagynus* cf. *cucumis* were from previous investigations (Chen et al. 2022; Xu et al. 2025). The 16S rDNA sequences (~ 1 kb) of methanogenic archaeal symbionts were amplified for *M. laminarius* and *M.* cf. *contortus* with the primers Ar109f (5ʹ-AMDGCTCAGTAACACGT-3ʹ) and Ar1490R (5ʹ-GGHTACCTTGTTACGACTT-3ʹ) (Takeshita et al. 2019). Another pair of primers, i.e., Archf2 (5ʹ-TTCYGGTTGATCCYGCCRGA-3ʹ) and Archr1386 (5ʹ-GCGGTGTGTGCAAGGAGC-3ʹ) (Skillman et al. 2004), was used for *Metopus* sp., *A.* cf. *cucumis*, *P.* cf. *narasimhamurtii* pop. 1, *P.* cf. *ramani*, and *P. marina*. Except for the annealing temperature (50 °C for Ar109f/Ar1490R, 61 °C for Arch f2/Arch r1386), the PCR reaction procedure was the same as that for 18S rDNA. All of the above PCR products were directly sequenced at Guangzhou Tianyi Huiyuan Gene Technology Co. Ltd, China, except for the methanogenic archaeal symbiont *M.* cf. *contortus* which was cloned into *pEASY*®-T1 vectors (TransGen, China) for sequencing.

16S rDNA V4 amplicons of methanogenic archaeal symbionts and free-living archaea in corresponding ciliate-free culture media (~ 400 bp) have been deposited in the NCBI Sequence Read Archive under BioProject PRJNA1134647. All new sequences have been submitted to GenBank with following accession numbers: PQ005718–PQ005723 (ciliate 18S rDNA sequences); PQ005725–PQ005731 (16S rDNA sequences of methanogenic archaeal symbionts, ~ 1 kb). The 18S rDNA sequence of *Apolagynus* cf. *cucumis* was obtained from Dr. Jiahui Xu, formerly of South China Normal University, by personal communication.

### Sources and analyses of sequences

A total of nine datasets were obtained for 16S rDNA V4 amplicons (~ 400 bp). Each dataset contained amplicon sequences of archaea in a given ciliate population and in a corresponding culture medium, respectively. Each dataset was denoised and clustered in an amplicon sequence variant (ASV) using the DADA2 (Callahan et al. 2016) function of QIIME 2 (Bolyen et al. 2019). Taxonomic assignments of representative ASV sequences were carried out using the Silva 138 database (Quast et al. 2013) and were classified using uclust in QIIME 1 (Caporaso et al. 2010) with default parameters. Non-archaeal representative sequences were removed before multiple rarefactions, and 900,195 (*Metopus* sp.) *–*112,925 (*Plagiopyla* cf. *narasimhamurtii* pop. 2) archaeal reads were kept for downstream analyses.

Eight datasets were used for phylogenetic analyses. Comparison of phylogenetic tree topologies between anaerobic ciliates and their endosymbiotic methanogens was performed based on Dataset 18S-host-135 and Dataset 16S-meth-135. (a) Dataset 18S-host-135: 18S rDNA sequences of 126 ciliates containing methanogenic endosymbionts from previous studies (Supplementary Table S2), as well as the nine newly sequenced ciliates from the present study. Among 134 ciliates in Supplementary Table S2, five were not included in Dataset 18S-host-135 for following reasons: 16S rDNA sequences of methanogenic endosymbionts for five ciliate species (*Plagiopyla ramani* KY563720, *Plagiopyla ovata* GOOSE1A OP186387, *Nyctotherus ovalis*, *Metopus es* and *Metopus* sp. LC062508) were unavailable or too short; 18S rDNA sequences of another two ciliates (*Metopus contortus* and *Urostomides striatus*) were unavailable; the geleiid karyorelictean ciliate (JF327426) was not included due to its long branch. (b) Dataset 16S-meth-135: 16S rDNA sequences of methanogenic endosymbionts hosted by ciliate species in Dataset 18S-host-135.

Phylogenetic relationships within the orders Methanomicrobiales and Methanobacteriales were inferred based on Dataset 16S-Mmic-132 and Dataset 16S-Mbac-78, respectively. (c) Dataset 16S-Mmic-132: 16S rDNA sequences of 94 endosymbiotic and 35 representative free-living methanogens belonging to order Methanomicrobiales, with *Methanolobus tindarius* (M59135), *Methanococcoides methylutens* (FR733669) and *Methanosarcina barkeri* (AJ012094) as the outgroup. (d) Dataset 16S-Mbac-78: 16S rDNA sequences of 42 endosymbiotic and 33 representative free-living methanogens belonging to order Methanobacteriales; outgroups were the same as Dataset 16S-Mmic-132. We also added Methanothermaceae (HE654004 and CP002278), *Methanosphaera* (HE582783 and CP000102), and *Methanothermobacter* (AY196660 and X15364) to Dataset 16S-Mbac-78 to include sequences covering two families of the order Methanobacteriales. For Datasets 16S-Mmic-132 and 16S-Mbac-78, 16S rDNA sequences of representative free-living methanogens were obtained using the following criteria: for each endosymbiotic methanogen genus, one representative sequence was used as a reference (NR112751 for *Methanoplanus*, MF074216 for *Methanocorpusculum*, LC466987 for *Methanoregula*, Z29436 for *Methanobacterium*, and AB118591 for *Methanobrevibacter*), and the top 1000 related sequences from GenBank were downloaded.

Phylogenetic relationships within the genera *Methanoregula*, *Methanocorpusculum*, *Methanobacterium*, and *Methanobrevibacter* were inferred based on Dataset 16S-Mregu-202, Dataset 16S-Mcor-104, Dataset 16S-Mbium-320, and Dataset 16S-Mbre-372, respectively. (e) Dataset 16S-Mregu-202 (with *Methanolinea mesophila* as the outgroup): 32 16S rDNA sequences of *Methanoregula* in 16S-Mmic-132 and 169 additional sequences. These additional sequences were obtained using the following criteria. All 16S rDNA sequences of free-living methanogens in the Silva 138 database classified as *Methanoregula* were downloaded. After removing short sequences (< 600 bp), the remaining sequences were clustered at 97% similarity using VSEARCH v2.15.0 (Rognes et al. 2016). (f) Dataset 16S-Mcor-104 (with three sequences of the family Methanocalculaceae as the outgroup): 67 16S rDNA sequences of *Methanocorpusculum* in 16S-Mmic-132 and 34 additional sequences. (g) Dataset 16S-Mbium-320 (with three sequences of *Methanobrevibacter* as the outgroup): 55 16S rDNA sequences of *Methanobrevibacter* in 16S-Mbac-78 and 262 additional sequences. (h) Dataset 16S-Mbre-372 (with five sequences of *Methanobacterium* as the outgroup): 25 16S rDNA sequences of *Methanobacterium* in 16S-Mbac-78 and 342 additional sequences. Additional sequences in datasets 16S-Mcor-104, 16S-Mbium-320, and 16S-Mbre-372 were obtained as for 16S-Mmic-132 above.

For phylogenetic analyses, each dataset was aligned using online software MAFFT v7 with default settings (Katoh and Standley 2013). The alignments were then trimmed with SeaView 4.7 (Gouy et al. 2010). IQ-TREE 2.1.4 (Nguyen et al. 2015) was used to calculate the best-fit nucleotide substitution models and maximum likelihood (ML) analyses were performed. Bayesian inference (BI) analyses were carried out using MrBayes v3.2.7a (Ronquist et al. 2012) with the best-fit model according to Akaike information criterion (AIC) in MrModeltest v2 (Nylander 2004). Four chains were set to run at least 1,000,000 generations and sampled every 100 generations. The first 2500 trees were discarded as burn-in. Only ML trees were constructed for four genera of methanogens (Dataset 16S-Mregu-202, Dataset 16S-Mcor-104, Dataset 16S-Mbium-320, and Dataset 16S-Mbre-372). Both ML and BI trees were inferred from Dataset 18S-host-135, Dataset 16S-meth-135, Dataset 16S-Mmic-132, and Dataset 16S-Mbac-78. Tree topologies were visualized in FigTree v1.4.4 (http://tree.bio.ed.ac.uk/software/figtree/), MEGA 11 (Tamura et al. 2021), and ITOL v4 (Letunic and Bork 2019).

### Fluorescence in situ hybridization (FISH)

Based on the amplification sequencing results, we verified the ciliate methanogenic endosymbionts by fluorescence in situ hybridization (FISH) as described by Omar et al. (2017). Briefly, ciliate cells were starved, washed several times, and fixed onto an adhesion microscope slide (Thermo, USA) with Bouin’s solution. After air-drying at room temperature, the slides were dehydrated in a graded series of 30%, 50%, 80%, and 100% ethanol, incubated with their corresponding probe and hybridization buffer at 46 °C for 3 h (Supplementary Table S3), and then washed with buffer at 48 °C for 30 min. Finally, the fluorescence signals were observed and imaged under an upright fluorescence microscope.

## Results

### Diversity of anaerobic ciliates and their methanogenic endosymbionts

Nine anaerobic ciliates with methanogenic endosymbionts were investigated, namely *Metopus* sp., *M.* cf. *contortus*, and *M. laminarius* (class Armophorea); *Plagiopyla marina*, *P.* cf. *narasimhamurtii* pop. 1, *P.* cf. *narasimhamurtii* pop. 2, and *P.* cf. *ramani* (class Plagiopylea); *Apolagynus* cf. *cucumis* (class Prostomatea); and *Thigmothrix strigosa* (class Muranotrichea) (Table 1). All nine showed strong blue fluorescence in violet light due to the presence of coenzyme F420, indicating that each one hosted a dense population of methanogenic endosymbionts (Fig. 1a2, b2, c2, d2, e2, f2, g2, h2, i2). The methanogenic endosymbionts of *M.* cf. *contortus* and *M. laminarius* were rod-shaped, while those of the other seven populations (viz. *Metopus* sp., *P. marina*, *P.* cf. *narasimhamurtii* pop. 1, *P.* cf. *narasimhamurtii* pop. 2, *P.* cf. *ramani*, *A.* cf. *cucumis*, and *T. strigosa*) were spherical.

**Table 1:** Classification of methanogenic endosymbionts inferred from results of 16S rDNA V4 amplicons and FISH

Ciliate host	Classification inferred from 16S rDNA V4 amplicon	Classification inferred from FISH
*Apolagynus* cf. *cucumis*	Genus *Methanocorpusculum*, order Methanomicrobiales	Order Methanomicrobiales
*Metopus* cf.* contortus*	Genus *Methanobacterium*, order Methanobacteriales	Order Methanobacteriales
*Metopus laminarius*	Genus *Methanobacterium*, order Methanobacteriales	Order Methanobacteriales
*Metopus* sp.	Genus *Methanocorpusculum*, order Methanomicrobiales	Order Methanomicrobiales
*Plagiopyla* cf. *narasimhamurtii* pop. 1	Genus *Methanocorpusculum*, order Methanomicrobiales	Order Methanomicrobiales
*Plagiopyla* cf. *narasimhamurtii* pop. 2*	Genus *Methanoplasma*, order Methanomicrobiales or genus *Methanocorpusculum*, order Methanomicrobiales	Order Methanomicrobiales
*Plagiopyla* cf. *ramani*	Genus *Methanocorpusculum*, order Methanomicrobiales	Order Methanomicrobiales
*Plagiopyla marina*	Genus *Methanocorpusculum*, order Methanomicrobiales	Order Methanomicrobiales
Thigmothrix strigose*	Genus *Methanocorpusculum*, order Methanomicrobiales	Order Methanomicrobiales

Note: Only if ASV is more abundant in ciliate cells than in corresponding culture media, it can be categorized as putative methanogenic symbiont

^*^The 16S rDNA sequences (~ 1 kb) were not available for *Plagiopyla* cf. *narasimhamurtii* pop. 2 and *Thigmothrix strigosa*, due to failure of PCR amplification

Comparisons of 16S rDNA V4 amplicons between intracellular archaea in ciliate cells and free-living archaea in corresponding culture media are useful for detecting potential methanogenic endosymbionts, although food contamination in cells and symbiont contamination in culture media could not be completely excluded. Our results showed that most of the methanogenic endosymbionts of *A.* cf. *cucumis*, *Metopus* sp., *P.* cf. *narasimhamurtii* pop.1, *P.* cf. *ramani*, *P. marina*, and *T. strigosa* might be *Methanocorpusculum* species (order Methanomicrobiales) (Supplementary Fig. S2; Table 1). Interestingly, *M.* cf. *contortus* and *M. laminarius* hosted methanogenic endosymbionts that were most similar to *Methanobacterium* species (order Methanobacteriales) (Supplementary Fig. S2; Table 1). The methanogenic endosymbionts of *P.* cf. *narasimhamurtii* pop. 2 could not be determined because Methanomassiliicoccales and Methanomicrobiales were abundant both within ciliate cells of this population and in its corresponding ciliate-free culture media (Supplementary Fig. S2; Table 1).

The Archaea-specific FISH-probe co-localized with the Methanomicrobiales-specific or Methanobacteriales-specific probe showing that the endosymbionts were distributed throughout the anaerobic ciliate cells (Fig. 1a3‒4, b3‒4, c3‒4, d3‒4, e3‒4, f3‒4, g3‒4, h3‒4, i3‒4). FISH results demonstrated that spherical endosymbionts of the order Methanomicrobiales were distributed throughout the cytoplasm of *Metopus* sp., *P.* cf. *narasimhamurtii* pop. 1, *P.* cf. *narasimhamurtii* pop. 2, *P. marina*, *P.* cf. *ramani*, *A.* cf. *cucumis*, and *T. strigosa*, and that the rod-shaped endosymbionts of *M. laminarius* and *M.* cf. *contortus* belonged to the order Methanobacteriales. TEM observations of *M.* cf. *contortus* revealed prokaryotic endosymbionts that were rod-shaped in longitudinal section and circular in cross-section, and that these were closely associated with MROs (Fig. 1J).

16S rDNA sequences (~ 1 kb) of methanogenic endosymbionts were newly obtained for seven ciliate populations, while those of *Plagiopyla* cf. *narasimhamurtii* pop. 2 and *Thigmothrix strigosa* were not available due to failure of PCR amplification. As expected, V4 regions of all these 16S rDNA sequences (~ 1 kb) are identical to corresponding ASV sequences of putative symbionts inferred from results of 16S rDNA V4 amplicons. Combining results of autofluorescence, 16S rDNA V4 amplicons, FISH, TEM, and the 16S rDNA sequences (~ 1 kb), methanogenic endosymbionts of *Metopus* sp., *P.* cf. *ramani*, *P.* cf. *narasimhamurtii* pop.1, *P.* cf. *narasimhamurtii* pop. 2, *P. marina*, *A.* cf. *cucumis*, and *T. strigosa* were identified as *Methanocorpusculum* species (order Methanomicrobiales), whereas those of *M.* cf. *contortus* and *M. laminarius* were identified as *Methanobacterium* species (order Methanobacteriales).

### Classifications of methanogenic endosymbionts

In total, methanogenic endosymbionts of nine anaerobic ciliates were newly sequenced (seven 16S rDNA sequences with a length of about 1.2 kb, two 16S rDNA sequences with a length of about 400 bp), and those of 134 others (310‒1953 bp) were obtained from published literature or public databases (Supplementary Table S2). The methanogenic endosymbionts mainly belonged to the orders Methanomicrobiales (67.83%) and Methanobacteriales (30.07%). Hence, 16S rDNA trees of these two orders were constructed to determine the classifications of the methanogenic endosymbionts observed.

**Fig. 2:**
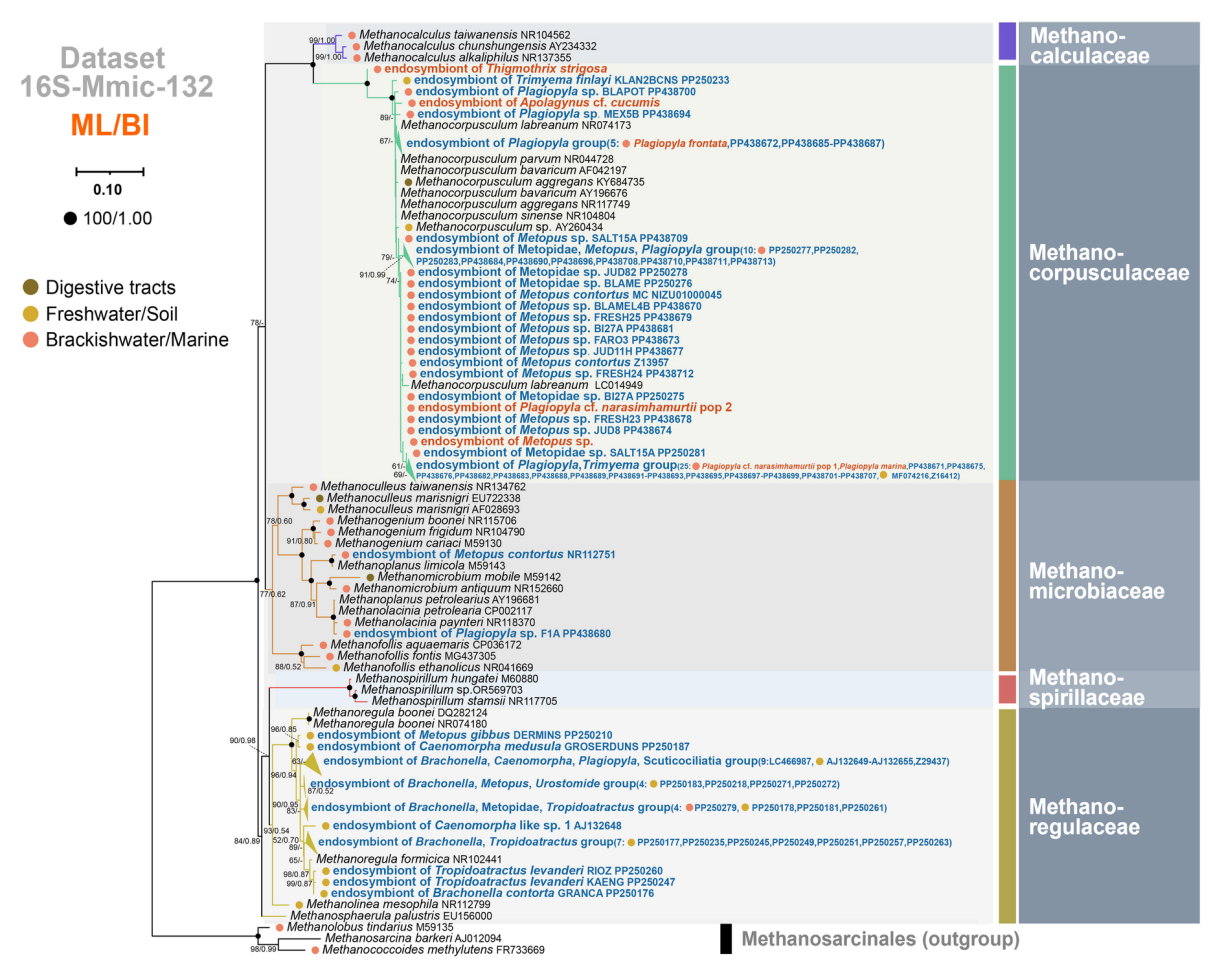
Phylogenetic trees of Methanomicrobiales inferred from Dataset 16S-Mmic-132. The sequences of endosymbiotic methanogens in anaerobic ciliates published in GenBank are shown in blue bold font, and the sequences newly obtained in this study are shown in red bold font. Habitats of free-living methanogens or ciliate hosts are labeled with colored dots (brown, digestive tract; orange, freshwater/soil; red, marine/brackish water). The numbers at nodes represent bootstrap values of maximum likelihood (ML) tree and posterior probabilities of Bayesian inference (BI) tree, respectively. Support values equal to or below 50%/0.50 are not shown, and full support values are labeled with black dots

The 16S rDNA trees of the order Methanomicrobiales comprised five families, namely Methanocalculaceae, Methanocorpusculaceae, Methanomicrobiaceae, Methanoregulaceae, and Methanospirillaceae (Fig. 2). None of the previously reported ciliate methanogenic endosymbionts grouped within the family Methanocalculaceae or Methanospirillaceae. Only the methanogenic endosymbionts of *Metopus contortus* NR112751 and *Plagiopyla* sp. PP438680 belonged to the family Methanomicrobiaceae. Most species of the family Methanoregulaceae grouped into a single clade, and methanogenic endosymbionts of 30 anaerobic ciliate populations fell into the family Methanoregulaceae and seemed to be most closely related to free-living *Methanoregula* species. These anaerobic ciliate populations were mostly sampled from freshwater or soil, and belonged to one of three classes, namely Armophorea, Plagiopylea, or Oligohymenophorea. The family Methanocorpusculaceae was monophyletic, and the phylogenetic trees suggested that the methanogenic endosymbionts of 62 anaerobic ciliate populations were members of this family. These anaerobic ciliate populations were mostly sampled from marine or brackish water, and belonged to one of four classes, i.e., Armophorea, Plagiopylea, Muranotrichea, or Prostomatea.

**Fig. 3:**
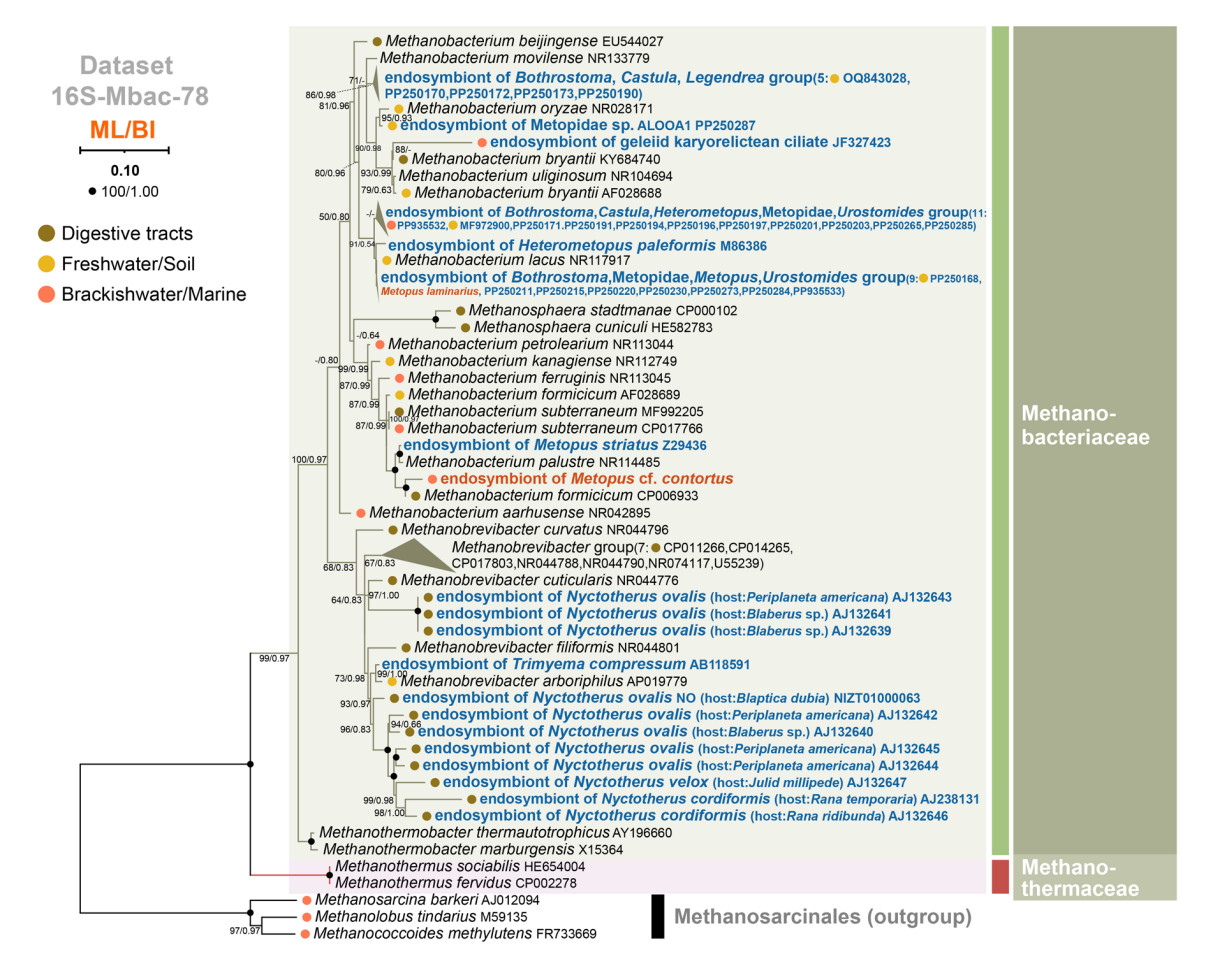
Phylogenetic trees of Methanobacteriales inferred from Dataset 16S-Mbac-78. The sequences of endosymbiotic methanogens in anaerobic ciliates published in GenBank are shown in blue bold font, and the sequences newly obtained in this study are shown in red bold font. Habitats of free-living methanogens or ciliate hosts are labeled with colored dots (brown, digestive tract; orange, freshwater/soil; red, marine/brackish water). The numbers at nodes represent bootstrap values of ML tree and posterior probabilities of BI tree, respectively. Support values equal to or below 50%/0.50 are not shown, and full support values are labeled with black dots

The 16S rDNA trees of the order Methanobacteriales revealed two well supported monophyletic families, viz. Methanobacteriaceae (99% ML, 0.97 BI) and Methanothermaceae (100% ML, 1.00 BI). All ciliate methanogenic endosymbionts grouped within the family Methanobacteriaceae (Fig. 3). Methanogenic endosymbionts of Metopidae sp., *Bothrostoma*, *Castula*, *Heterometopus*, *Metopus*, and *Urostomides* in the class Armophorea, as well as those of *Legendrea* in the classes Litostomatea and of the geleiid in the class Karyorelictea, grouped within the *Methanobacterium* + *Methanosphaera* clade. In contrast, methanogenic endosymbionts of *Nyctotherus* and *Trimyema* grouped within the *Methanobrevibacter* clade (Fig. 3).

In both above-mentioned trees (Figs. 2, 3), most endosymbionts of free-living and endocommensal anaerobic ciliates grouped with free-living methanogens collected from the same respective habitats. To better clarify the sources of methanogenic endosymbionts, environmental sequences were added to reconstructed phylogenetic trees of *Methanobrevibacter*, *Methanobacterium*, *Methanoregula* and *Methanocorpusculum* (Supplementary Figs. S3–S6). In each case, the endosymbionts of anaerobic ciliates usually formed several clades and then were sister to free-living methanogens. Few endosymbionts directly grouped with free-living methanogens and were scattered in these trees (Figs. 2, 3; Supplementary Figs. S3–S6). This finding supports the assertion that most methanogenic endosymbionts are sister to their free-living counterparts collected from the same habitat as the ciliate hosts of the former. In addition, it seemed that the branches of methanogenic endosymbionts of free-living armophoreans were shorter than those of their free-living relatives, while branches of methanogenic endosymbionts of endocommensal armophoreans were of similar length to those of their free-living counterparts (Supplementary Figs. S3–S6).

### Phylogenies of armophorean and plagiopylean ciliates and their methanogenic endosymbionts

**Fig. 4:**
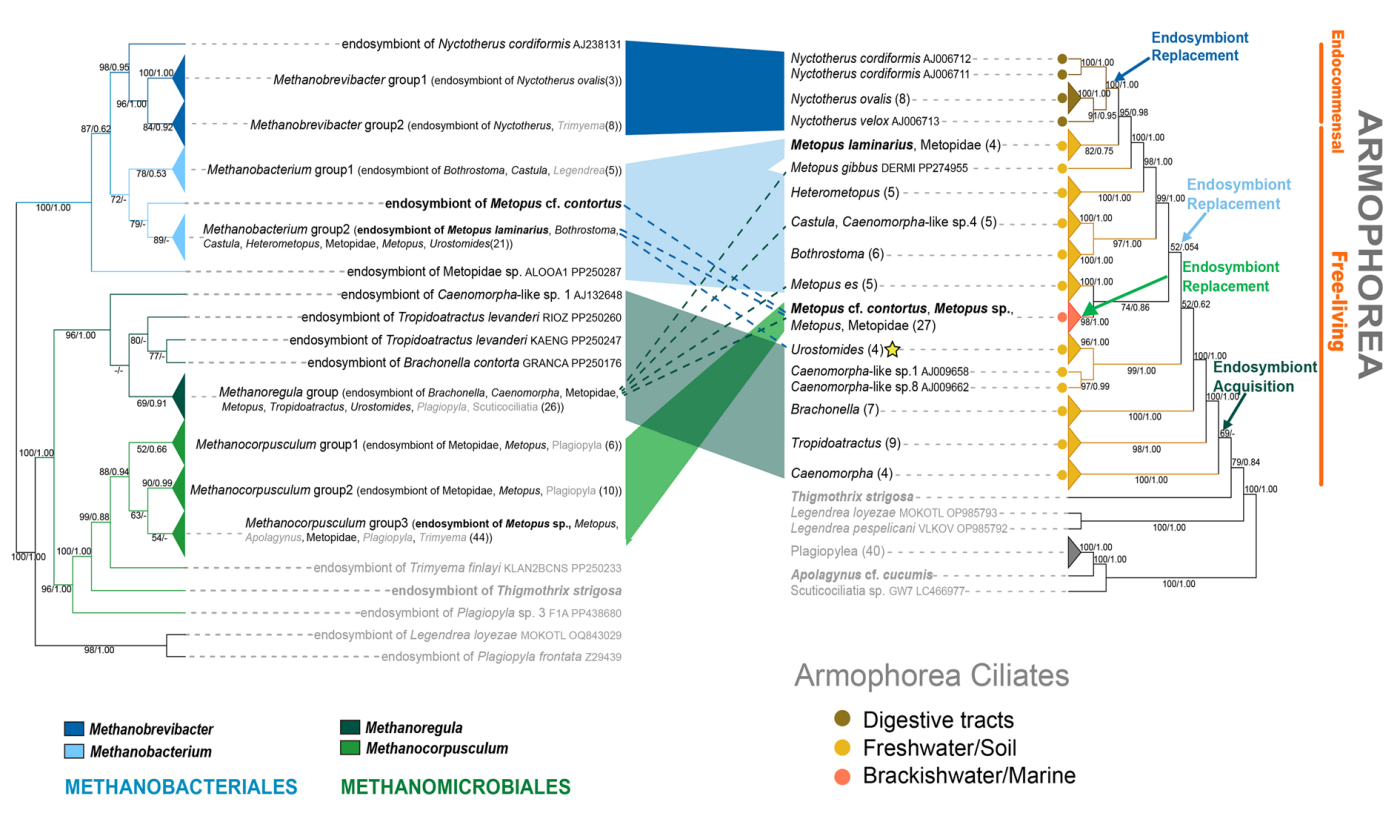
Comparison of phylogenetic tree topologies between ciliates in class Armophorea (right) and their endosymbiotic methanogens (left) (detailed information in Supplementary Fig. S7). The relationships of the host ciliates and their methanogenic endosymbionts are connected by different color blocks. The dash line represents a possible recent replacement event of endosymbiotic methanogens, the star represents two host ciliate species in the same group having possible recent replacement events. Habitats of ciliate hosts are labeled with colored dots (brown, digestive tract; orange, freshwater/soil; red, marine/brackish water). Newly sequenced ciliates and their endosymbiotic methanogens in this study are shown in bold black. The numbers at nodes represent bootstrap values of ML tree and posterior probabilities of BI tree, respectively. Support values equal to or below 50% are not shown

**Fig. 5:**
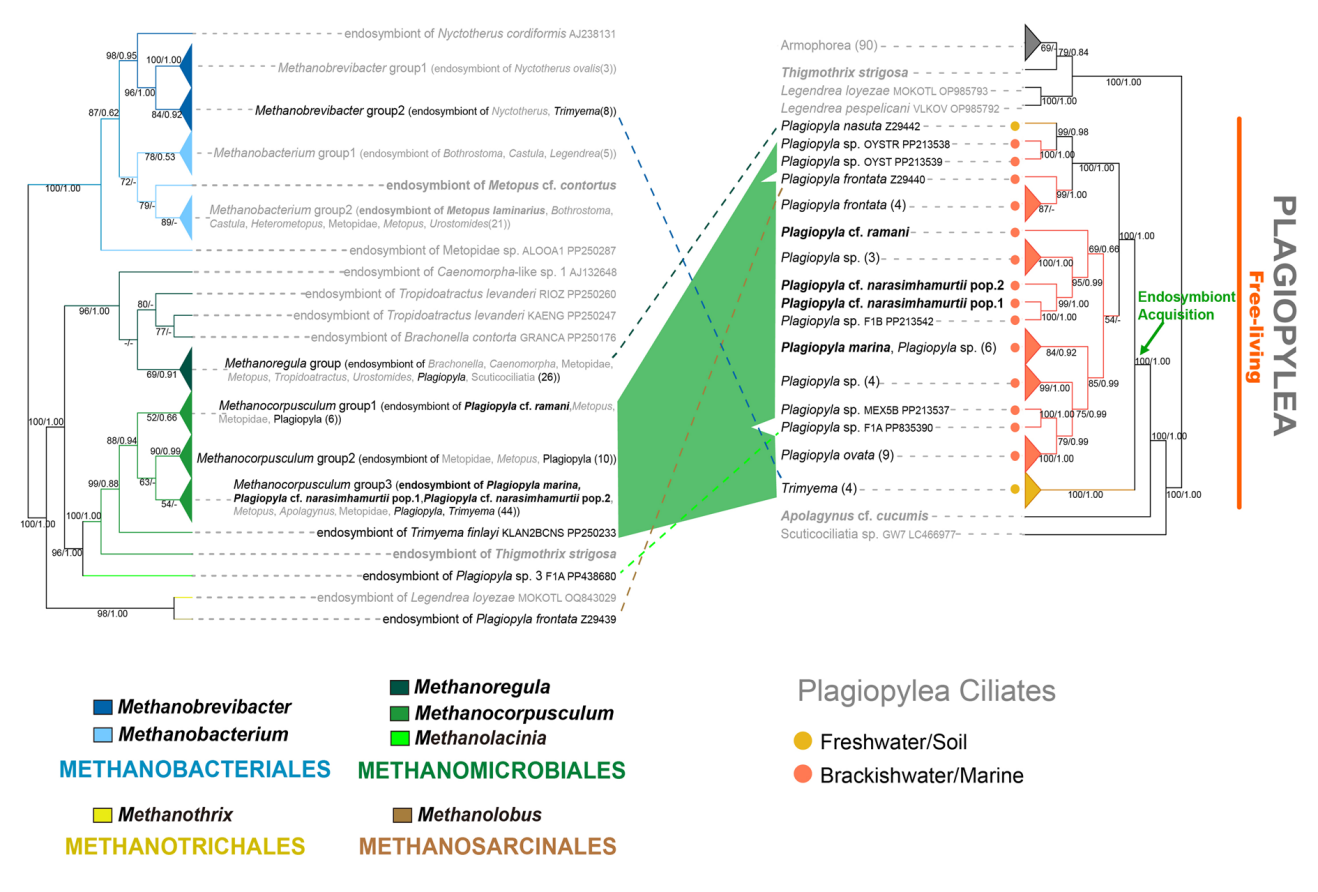
Comparison of phylogenetic tree topologies between ciliates in class Plagiopylea (right) and their endosymbiotic methanogens (left) (detailed information in Supplementary Fig. S7). The relationships of the host ciliates and their methanogenic endosymbionts are connected by different color blocks, and the dash line represents possible recent replacement events of endosymbiotic methanogens. Habitats of ciliate hosts are labeled with colored dots (orange, freshwater/soil; red, marine/brackish water). Newly sequenced ciliates and their endosymbiotic methanogens in this study are shown in bold black. The numbers at nodes represent bootstrap values of ML tree and posterior probabilities of BI tree, respectively. Support values equal to or below 50% are not shown

Phylogenetic comparisons between host ciliates (18S-host-135) and their methanogenic endosymbionts (16S-meth-135) showed that methanogenic endosymbionts of endocommensal species in class Armophorea, free-living species in class Armophorea, and species in class Plagiopylea had different evolutionary histories (Figs. 4, 5; Supplementary Fig. S7).

Phylogenies of endocommensal armophorean ciliates and of their methanogenic endosymbionts matched each other well (Fig. 4; Supplementary Fig. S7). In 18S-host-135 trees, the endocommensal armophorean ciliates were monophyletic with full support (100% ML, 1.00 BI) (Fig. 4; Supplementary Fig. S7). Similar to the topology of 18S-host-135 trees, the methanogenic endosymbionts of these endocommensal armophorean ciliates and the endosymbiont of *Trimyema compressum* AB118591 formed a clade and were species of *Methanobrevibacter* (order Methanobacteriales) (Fig. 4; Supplementary Fig. S7).

Comparison of 18S-host-135 and 16S-meth-135 trees showed that parts of the free-living armophorean ciliate tree and their methanogenic endosymbiont tree were congruent (Fig. 4; Supplementary Fig. S7). In 18S-host-135 trees, free-living armophorean ciliates were polyphyletic, and formed a highly supported clade (95% ML, 0.98 BI) with endocommensal species. Interestingly, 27 *Metopus* and Metopidae populations sampled from marine/brackish water grouped together with high support (98% ML, 1.00 BI). Most of their endosymbionts were *Methanocorpusculum* species (order Methanomicrobiales). By contrast, most endosymbionts of the freshwater/soil armophoreans occupying basal and crown positions belonged to the genera *Methanoregula* (order Methanomicrobiales) and *Methanobacterium* (order Methanobacteriales), respectively. Only eight populations of free-living armophoreans and their endosymbionts did not follow this pattern (Fig. 4; Supplementary Fig. S7).

Similar to free-living armophorean ciliates, a partial match between the phylogenies of free-living plagiopyleans and their methanogenic endosymbionts was revealed (Fig. 5; Supplementary Fig. S7). In 18S-host-135 trees, the free-living plagiopyleans were monophyletic and comprised the *Trimyema* and *Plagiopyla* clades. *Plagiopyla nasuta* (Z29442) and all *Trimyema* populations were collected from freshwater/soil habitats, whereas other *Plagiopyla* populations were collected from marine/brackish water. Most endosymbionts of plagiopyleans grouped within the *Methanocorpusculum* clade (order Methanobacteriales) (Fig. 5; Supplementary Fig. S7). Endosymbionts of three *Plagiopyla* populations and one *Trimyema* population were scattered within the genera *Methanoregula* (order Methanomicrobiales), *Methanolacinia* (order Methanomicrobiales), *Methanolobus* (order Methanosarcinales), and *Methanobrevibacter* (order Methanobacteriales), respectively.

## Discussion

### Methanogenic endosymbionts are universal in diverse anaerobic ciliates and their associations tend to be specific rather than random

**Fig. 6:**
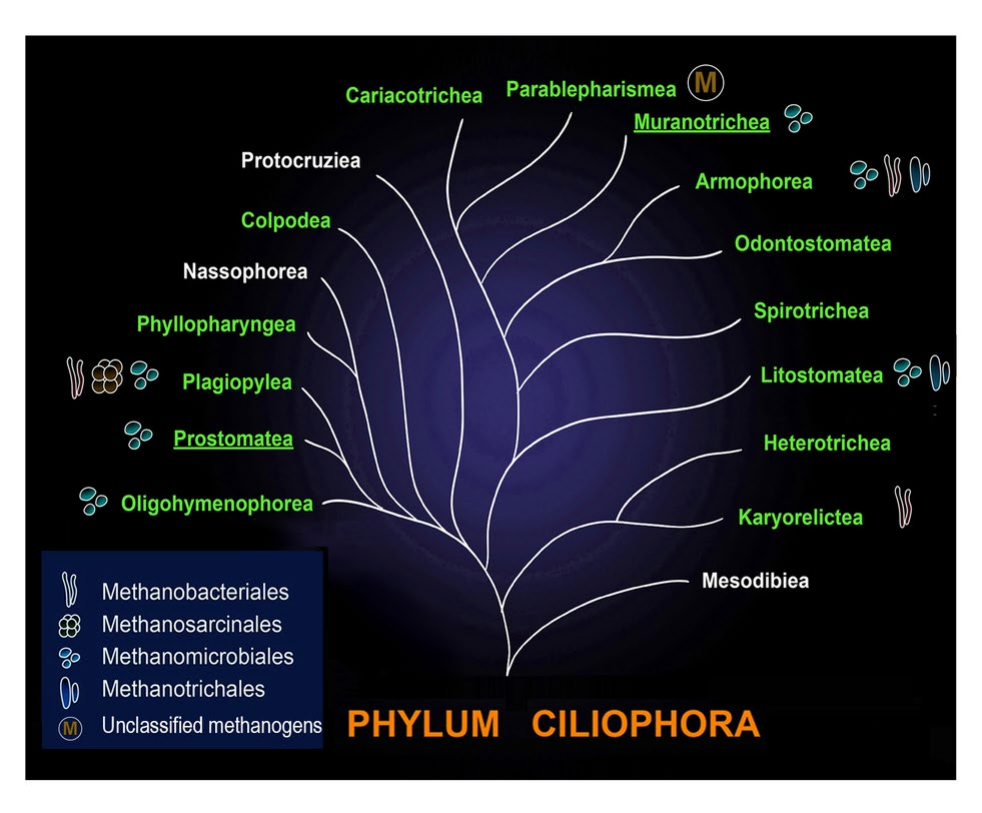
General overview of ciliate classes and their endosymbiotic methanogens. Phylogenetic relationships among ciliate classes are modified from Chen et al. (2022). Green font indicates ciliate classes that contain anaerobic species. Ciliate classes reported here with endosymbiotic methanogens for the first time are underlined

Methanogenic endosymbionts are common in diverse anaerobic ciliate classes (Fig. 6). Methanogenic endosymbionts have been reported in at least six ciliate classes, namely Armophorea, Karyorelictea, Litostomatea, Oligohymenophorea, Parablepharismea, and Plagiopylea (Supplementary Table S2; Beinart et al. 2018; Clarke et al. 1993; Edgcomb et al. 2011; Embley and Finlay 1994; Embley et al. 1992a, b; Finlay et al. 1993; Gijzen et al. 1991; Hirakata et al. 2015; Lewis et al. 2018; Li et al. 2023b; Lind et al. 2018; Méndez-Sánchez et al. 2024; Narayanan et al. 2009; Nitla et al. 2019; Pomahač et al. 2023; Rotterová et al. 2020; Schrecengost et al. 2024; Shinzato et al. 2007; Takeshita et al. 2019; van Bruggen et al. 1984, 1986; van Hoek et al. 2000; Zhuang et al. 2022). However, the classification of methanogenic endosymbionts of Parablepharismea is not clear (Rotterová et al. 2020), therefore these are not included in Supplementary Table S2. The present study is the first to report that anaerobic ciliates in the classes Muranotrichea (represented by *Thigmothrix strigosa*) and Prostomatea (represented by *Apolagynus* cf. *cucumis*) host methanogenic endosymbionts (Fig. 1).

Associations between anaerobic ciliates and their methanogenic endosymbionts are mutualistic. For ciliates, the faster removal of undesirable hydrogen and other metabolic waste by methanogenic symbionts improves the rate and efficiency of ciliate metabolism (Rotterová et al. 2020). It has also been demonstrated that some anaerobic ciliates grow much slower when their methanogenic endosymbionts are inhibited (Fenchel and Finlay 1991; Finlay and Fenchel 1992). On the other hand, ciliate hosts provide their methanogenic endosymbionts with stable, grazer-free environments, and supply them with hydrogen for generating energy (Jones et al. 1987; van Bruggen et al. 1983). Previous studies have reported that anaerobic ciliates usually move rapidly and tend to have a large cell size, thus requiring the increased metabolic efficiency imparted by symbionts since the energy metabolism of their MROs alone is insufficient (Rotterová et al. 2020). This is probably the reason that the presence of methanogenic endosymbionts is so common for diverse anaerobic ciliates.

It has been demonstrated that endosymbiotic methanogens tend to be associated with particular host ciliate species. Previous studies showed that 16S rDNA sequences of ciliate methanogenic endosymbionts were related to, but not identical with, free-living methanogens (Fenchel and Finlay 2010; Hackstein 2018; Hirakata et al. 2015; van Hoek et al. 2000). With more available 16S rDNA sequences of free-living methanogens including uncultured ones, our investigation drew the same conclusion (Figs. 2, 3; Supplementary Figs. S3–S6). To explain the evolutionary acquisition of endosymbiotic methanogens by ciliates, it has been posited that anaerobic ciliates graze free-living methanogens from their surrounding environments as a food source, and that a few methanogens occasionally escape digestion and survive in the ciliate cytoplasm as endosymbionts (Dziallas et al. 2012; Hackstein 2018; Rotterová et al. 2020). Using amplicon sequencing, a recent investigation and the present study compared archaeal compositions in ciliate cells with those in ciliate-free culture media, and in both cases, the findings argued against the ciliate/endosymbiont association being random (Table 1; Méndez-Sánchez et al. 2024). Furthermore, the same endosymbiont species in two *Trimyema finlayi* populations, which were collected from very distant locations, supported the spatial and temporal stability of the association between host ciliates and their methanogenic endosymbionts (Lewis et al. 2018). Similar patterns of symbiont fidelity were also observed in geographically distant populations of *Metopus* sp. and *Plagiopyla ovata* (Schrecengost et al. 2024). Furthermore, by culturing co-occurring anaerobic ciliate species harboring different methanogenic symbionts together over a short time, the results showed that the relationships between ciliate hosts and their corresponding methanogenic endosymbionts are stable (Méndez-Sánchez et al. 2024).

Our analyses showed that anaerobic ciliates were associated with specific, rather than random, methanogenic endosymbionts. Hitherto, 134 ciliate populations and their methanogenic endosymbionts with order classifications have been reported (Supplementary Table S2). The present study has added data for another nine ciliate populations (Table 1). Analyses of all these data showed that among the methanogenic endosymbionts, the orders Methanomicrobiales (67.83%) and Methanobacteriales (30.07%) were the dominant groups, whereas the orders Methanosarcinales (0.70%) and Methanotrichales (1.40%) were rarely reported (Fig. 6; Supplementary Table S2). There are nine orders of methanogens, viz. Methanococcales, Methanopyrales, Methanobacteriales, Methanomicrobiales, Methanocellales, Methanonatronarchaeales, Methanosarcinales, Methanomassiliicoccales, and Methanotrichales (Akinyemi et al. 2025; Lyu et al. 2018). However, only four genera (*Methanocorpusculum*, *Methanolacinia*, *Methanoregula*, and *Methanoplanus*) in the order Methanomicrobiales, two genera (*Methanobacterium* and *Methanobrevibacter*) in the order Methanobacteriales, *Methanothrix* in the order Methanotrichales, and *Methanolobus* in order Methanosarcinales have been reported as ciliate endosymbionts (Table 1; Supplementary Table S2). Methanogens produce methane by five pathways, i.e., the hydrogenotrophic, acetoclastic, methylotrophic, methoxydotrophic, and alkylotrophic pathways (Mayumi et al. 2016; Zhang et al. 2020; Zhou et al. 2022). Among eight endosymbiotic genera, only *Methanothrix* (order *Methanotrichales*) and *Methanolobus* (order Methanosarcinales) are acetoclastic and methylotrophic methanogens, respectively, whereas the other six genera are hydrogenotrophs utilizing H_2_ and CO_2_ to produce methane (Guo et al. 2012; Zhang et al. 2020). It has been suggested that the armophorean ciliate *Metopus es* produces acetate by intracellular digestion of a complex carbon source, and its endosymbiotic methanogens, which were originally reported as *Methanosaeta* (later renamed *Methanothrix* in the order Methanotrichales), converts acetate to methane (Narayanan et al. 2009; Rosenberg et al. 2014). It is not surprising that most methanogenic endosymbionts are hydrogenotrophs as one of their roles is to reduce the partial pressure of hydrogen (Hackstein 2018; Méndez-Sánchez et al. 2024; Rotterová et al. 2022). At least 22 methanogenic genera have been reported to be hydrogenotrophs (Rosenberg et al. 2014; Zhang et al. 2020), but it is unclear whether any apart from the six reported here might be candidate methanogenic endosymbionts of anaerobic ciliates.

By comparing genomes of methanogenic endosymbionts inhabiting ciliates of the class Armophorea with their closest free-living counterparts, researchers have begun to reveal the molecular mechanisms by which methanogenic endosymbionts adapted to an intracellular lifestyle (Beinart et al. 2018; Lind et al. 2018). The methanogenic endosymbionts in question are: *Methanocorpusculum* sp. MCE (order Methanomicrobiales) within *Metopus contortus*; *Methanobrevibacter* sp. NOE (order Methanobacteriales) within *Nyctotherus ovalis*; and *Methanobacterium* sp. CSS (order Methanobacteriales) within *Heterometopus* sp. The loss of genes involved in amino acid biosynthesis, a large number of pseudogenes, and some genes potentially encoding secreted proteins, were observed in the former two endosymbionts, whereas an alternative strategy towards endosymbiosis was revealed in *Methanobacterium* sp. CSS (Beinart et al. 2018; Lind et al. 2018). In the latter, no significant reduction in genome size was observed, while several unique genes related to cross-membrane transport, adhesion, or surface proteins are thought to play an important role in interactions with the host cell (Beinart et al. 2018). It is likely that several different strategies have been adopted for establishing stable endosymbiotic associations between intracellular methanogens and their ciliate hosts, considering that four methanogenic orders inhabiting in six ciliate classes have been reported (Figs. 2–6; Supplementary Fig. S7, Table S2).

### Methanogenic endosymbionts of different anaerobic ciliate groups exhibit distinct origins and mixed-model transmissions

**Fig. 7:**
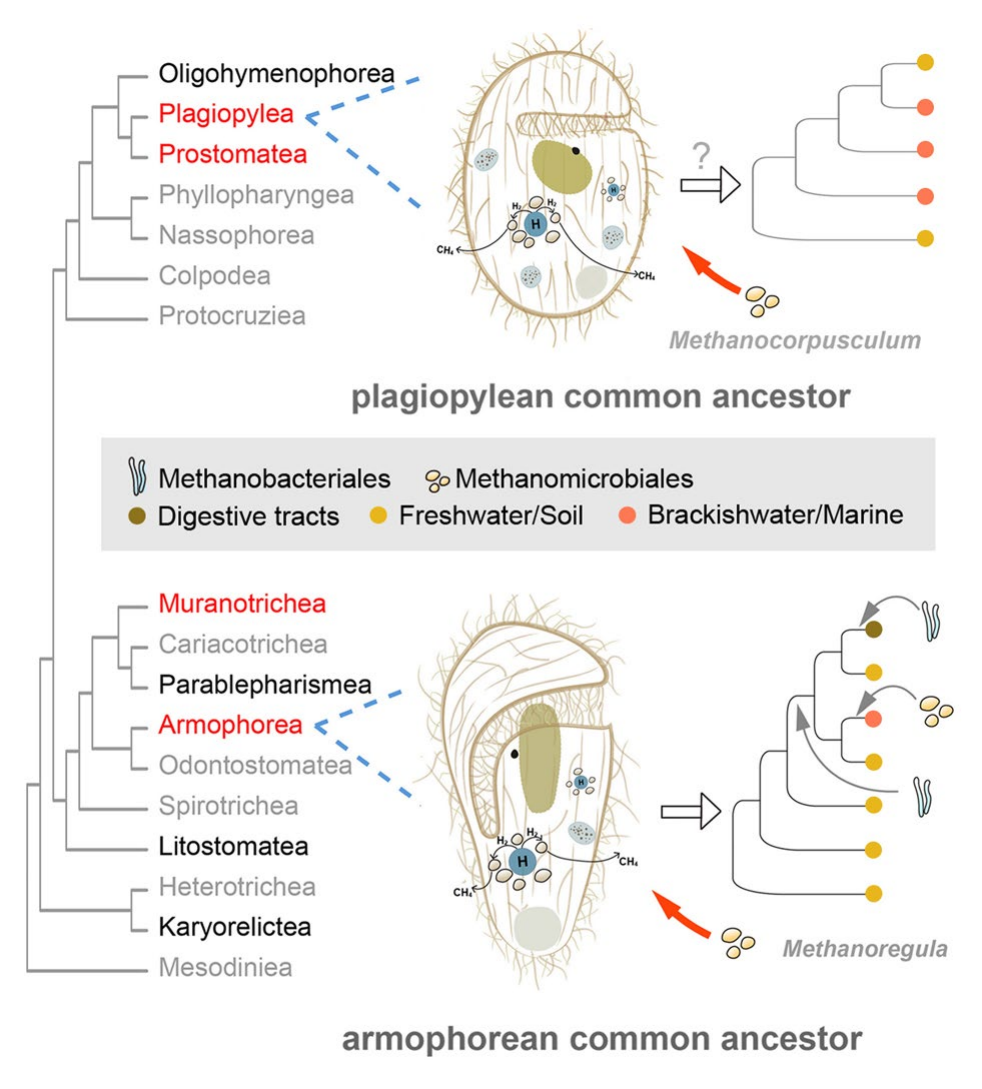
Independent origins and mixed-model transmissions are revealed for endosymbiotic methanogens of anaerobic ciliate classes Plagiopylea and Armophorea. Phylogenetic relationships among ciliate classes are modified from Chen et al. (2022). Black and red fonts indicate ciliate classes that include examples hosting methanogenic endosymbionts in their cytoplasm. The ciliate classes investigated in the present study are in red font. “?” means that acquisition and replacements of plagiopylean endosymbionts are still uncertain due to only endosymbionts of limited plagiopylean genera from specific habitats having been studied

Clarifying the evolution of associations between methanogenic endosymbionts and their ciliate hosts is a necessary precondition for illustrating mechanisms by which endosymbioses between these partners were established. Previous investigations assumed that transitions from mitochondria to MROs of these anaerobic ciliates occurred multiple times, and that methanogenic endosymbionts were only acquired after these events (Hackstein and Vogels 1997). Among the eight ciliate classes now known to have species containing methanogenic endosymbionts, four (Muranotrichea, Plagiopylea, Parablepharismea, and Armophorea) include only obligate or facultative anaerobes, whereas the other four (Litostomatea, Prostomatea, Oligohymenophorea, and Karyorelictea) are predominantly aerobic with only a small proportion that are obligately or facultatively anaerobic (Chen et al. 2022; Rotterová et al. 2022). There is growing evidence that methanogenic endosymbionts were acquired multiple times by their ciliate hosts. Phylogenetic trees of anaerobic ciliates, as well as functional patterns of their MROs, strongly suggest that MROs of different groups derive from independent transitions from mitochondria (e.g., Chen et al. 2022; Hirakata et al. 2015; Lewis et al. 2019; Li et al. 2023a; Rotterová et al. 2020; Su et al. 2023; van Hoek et al. 2000). Hence, it is highly likely that different anaerobic ciliate groups acquired methanogenic endosymbionts independently multiple times during their evolutionary history, because MROs and methanogenic endosymbionts are usually closely associated in ciliates, and the presence of both appears to be crucial for adaptation of ciliates to anoxia (Rotterová et al. 2020). Though strict co-diversification between anaerobic ciliates and their methanogenic endosymbionts was not observed in the present (Figs. 4, 5; Supplementary Fig. S7) or previous investigations (Méndez-Sánchez et al. 2024; Schrecengost et al. 2024; van Hoek et al. 2000), our analyses suggest that the anaerobic classes Plagiopylea and Armophorea might have harbored *Methanocorpusculum* and *Methanoregula,* respectively, as methanogenic endosymbionts at the beginning of their evolution (Figs. 4, 5, 7).

It is noteworthy that endosymbiont replacements occurred during the evolution of endosymbiotic associations between methanogens and their ciliate hosts. Members of the anaerobic class Armophorea, with a common ancestry of their MROs, were thought to host a common ancestral methanogenic endosymbiont (Fig. 7; van Hoek et al. 2000). However, armophorean species host methanogens belonging to two orders, i.e., Methanomicrobiales and Methanobacteriales (Fig. 4; Supplementary Fig. S7; Table 1; Supplementary Table S2; Beinart et al. 2018; Embley et al. 1992a, b; Gijzen et al. 1991; Hirakata et al. 2015; Lind et al. 2018; Narayanan et al. 2009; van Bruggen et al. 1984, 1986). A similar situation for hosting a common ancestral methanogenic endosymbiont also occurred in the anaerobic class Plagiopylea (Fig. 5; Supplementary Fig. S7; Table 1; Supplementary Table S2; Embley and Finlay 1994; Finlay et al. 1993; Lewis et al. 2018; Nitla et al. 2019; Shinzato et al. 2007). Furthermore, different methanogens have been detected in different populations of the same ciliate species. *Methanoregula* sp. and *Methanobacterium* sp. (order Methanobacteriales) are localized within the cells of four populations of *Metopus es*, respectively (Fig. 4; Supplementary Fig. S7, Table S2; Méndez-Sánchez et al. 2024). And *Methanocorpusculum* sp. (order Methanomicrobiales) and *Methanolobus* sp. (order Methanosarcinales) are localized within the cells of five different populations of *Plagiopyla frontata*, respectively (Fig. 5; Supplementary Fig. S7, Table S2; Embley and Finlay 1994). These findings suggest that strict co-diversification is not present between ciliates and their methanogenic endosymbionts, as revealed in aerobic ciliates of the genus *Euplotes* (class Spirotrichea) and their bacterial endosymbionts (Boscaro et al. 2019). However, methanogenic endosymbionts of anaerobic ciliates usually exhibit vertical transmission and specificity of association with their hosts to some degree (Lind et al. 2018; Rotterová et al. 2022). For instance, *Plagiopyla frontata* was observed synchronizing the division of methanogenic endosymbionts with its life cycle (Fenchel and Finlay 1991). Two populations of *Trimyema finlayi* were shown to host the same methanogenic endosymbiont species (Lewis et al. 2018), whereas different *Caenomorpha*-like species collected from the same site possess distinct endosymbiotic methanogen species (van Hoek et al. 2000). These findings indicate that multiple endosymbiont replacements have occurred during the vertical transmission of methanogenic endosymbionts. The close phylogenetic relationships between methanogenic endosymbionts and their free-living counterparts support the assertion that methanogens were acquired by their ciliate hosts from environmental sources (Figs. 2, 3; Supplementary Figs. S3–S6). One reasonable explanation for endosymbiont replacements is that transmission bottlenecks might have occurred during vertical transmission due to the purifying activity of natural selection (Bright and Bulgheresi 2010). On the other hand, methanogens are abundant in anaerobic environments, and it is likely that a few occasionally escape digestion and survive in the cytoplasm following their ingestion by anaerobic ciliates (Hackstein 2018; Husnik et al. 2021; van Hoek et al. 2000). Therefore, endosymbiont replacements probably facilitated transitions of ciliate hosts to new environments. Endosymbiont replacement events have also been reported for endosymbionts of some other ciliates. For instance, pre-existing endosymbiotic bacteria of *Euplotes* could be replaced by other bacteria and even ones belonging to different classes (Boscaro et al. 2017, 2018).

As expected, the present study revealed an independent origin and a mixed-model transmission for the anaerobic class Plagiopylea (Figs. 5, 7). Most plagiopylean ciliates, including marine *Plagiopyla* species of the family Plagiopylidae and freshwater/soil *Trimyema* species of the family Trimyemidae, were found to host *Methanocorpusculum* species (order Methanomicrobiales) as endosymbionts. Individual species of *Methanobrevibacter* (order Methanobacteriales), *Methanoregula* (order Methanomicrobiales), *Methanolacinia* (order Methanomicrobiales), and *Methanolobus* (order Methanosarcinales) were detected as endosymbionts in *Trimyema compressum*, *Plagiopyla nasuta*, *Plagiopyla* sp. F1A, and *Plagiopyla frontata*, respectively (Fig. 5; Supplementary Fig. S7; Table 1; Supplementary Table S2). Goosen et al. (1990) showed that *Trimyema compressum* cells can lose their methanogenic endosymbionts under culture conditions, supporting the assertion that the symbiotic relationship is not stable and that the endosymbionts might have been very recently acquired by the ciliate. Hence, we posit that the last common ancestor of Plagiopylea harbored *Methanocorpusculum* species as methanogenic endosymbionts and that during the evolution of this anaerobic ciliate class, *Methanobrevibacter*, *Methanoregula*, or *Methanolobus* were acquired by some plagiopyleans as endosymbiotic substitutes. However, conclusions about the origin and transmission of methanogenic endosymbionts of the class Plagiopylea might be arbitrary, considering that only marine *Plagiopyla* species and freshwater/soil *Trimyema* species were included in the present analyses. In future, methanogenic endosymbionts of freshwater/soil *Plagiopyla* species, marine *Trimyema* species, and other plagiopylean genera need to be studied to test this hypothesis.

Similarly, phylogenies of the anaerobic class Armophorea and their methanogenic endosymbionts did not match well, probably due to endosymbiont replacements (Figs. 4, 7; Supplementary Fig. S7; Hackstein 2018; Hirakata et al. 2015; van Hoek et al. 2000). Mapping of ecological niches onto phylogenetic trees indicated that the last common ancestor of armophoreans was probably an anaerobic free-living freshwater or soil species (Fig. 4; Supplementary Fig. S7; Li et al. 2023a; van Hoek et al. 2000). During the evolutionary history of Armophorea, ecological transitions to marine, brackish water and digestive tract niches occurred for some species (Fig. 4; Supplementary Fig. S7; Li et al. 2023a; van Hoek et al. 2000). A recent investigation with dense sampling demonstrated some armophorean subclades showed clear preferences for specific methanogenic genera (Méndez-Sánchez et al. 2024). Though placements of some armophorean subclades are slightly variable and not robustly supported in our trees (Figs. 4, 5) and previous small subunit ribosomal DNA (SSU rDNA) trees (Li et al. 2023a; Méndez-Sánchez et al. 2024), their general clustering patterns are similar. Our further insights into the phylogeny of ciliates and methanogens showed that most freshwater/soil ciliates in basal armophorean assemblages host species belonging to *Methanoregula* (order Methanomicrobiales), and that marine/brackish water armophoreans and other freshwater/soil species mostly host species belonging to *Methanocorpusculum* (order Methanomicrobiales) and *Methanobacterium* (order Methanobacteriales), respectively (Fig. 4; Supplementary Fig. S7; Table 1; Supplementary Table S2). By contrast, endocommensal species, which form a monophyletic group and occupy the crown position within the Armophorea, host species of *Methanobrevibacter* (order Methanobacteriales) (Fig. 4; Supplementary Fig. S7, Table S2). This indicates that the last common ancestor of the class Armophorea harbored *Methanoregula* species as methanogenic endosymbionts, thus facilitating its survival in anaerobic freshwater/soil environments. Subsequently, at least three endosymbiont replacement events (*Methanocorpusculum*, *Methanobacterium*, or *Methanobrevibacter* as substitutes for *Methanoregula*) occurred, possibly facilitated by transitions to new habitats, ecological shifts to different niches, species radiation of ciliate hosts, or vertical transmission bottlenecks of endosymbionts. It is noteworthy that most endosymbiotic *Methanocorpusculum* species that are localized in the cytoplasm of marine species of *Metopus* and other metopids are more closely related to endosymbionts of some anaerobic marine plagiopyleans than to free-living methanogens (Supplementary Fig. S4). Probably, the common ancestor of these marine *Metopus* and other metopid species acquired novel endosymbionts by host switches rather than from environmental sources. To facilitate their survival in digestive tracts, armophoreans probably acquired their methanogenic endosymbionts from this particular environment. Previous phylogenetic trees showed that methanogenic endosymbionts of free-living and endocommensal armophoreans cluster with free-living methanogens from external environments and digestive tracts, respectively, although based on a relatively small sample size (Hackstein 2018; Hirakata et al. 2015; van Hoek et al. 2000). With broader sampling of free-living methanogens, the present phylogenetic analyses of methanogens showed similar results (Supplementary Figs. S3–S6). The most parsimonious hypothesis is that some cells of a particular methanogenic species were taken up by a ciliate host as endosymbionts, whereas others survived as free-living methanogens. Then endosymbiotic methanogens and free-living methanogens evolved separately. Furthermore, the fact that the phylogenies of armophoreans and their methanogenic endosymbionts are generally congruent is inconsistent with the notion that frequent endosymbiont replacements were acquired from environmental sources (Fig. 4; Supplementary Fig. S7). Nevertheless, we cannot exclude the possibility that very recent and unstable endosymbiont replacement events might have happened at species or population level for ciliate hosts, e.g., endosymbionts of *Metopus es* (Fig. 4; Supplementary Figs. S5, S7) (Méndez-Sánchez et al. 2024). Further insights into phylogenetic trees of methanogens showed that the branches of methanogenic endosymbionts of free-living armophoreans are shorter than those of their free-living relatives, whereas branches of methanogenic endosymbionts of endocommensal armophoreans were of similar length to those of their free-living counterparts (Supplementary Figs. S3–S6). This might be because compared to external environments, intracellular environments and digestive tracts provide methanogens with stable and nutrient-rich conditions, thereby reducing their mutation rates (Bright and Bulgheresi 2010). In summary, armophoreans probably acquired methanogenic endosymbionts at the beginning of their evolution and although these were mostly retained, a few endosymbiont replacement events subsequently occurred, so the mode of endosymbiont transmission is mixed (Fig. 4; Supplementary Fig. S7; Hackstein 2018; van Hoek et al. 2000).

Among the ciliate classes Oligohymenophorea, Karyorelictea, Litostomatea, Prostomatea, and Muranotrichea, only one or two species hosting methanogenic endosymbionts were included in the phylogenetic trees (Supplementary Fig. S7). At present, it is impossible to determine the origins or modes of transmission of their methanogenic endosymbionts. Notably, the prostomatean species *Apolagynus* cf. *cucumis* and most plagiopyleans host *Methanocorpusculum* as methanogenic endosymbionts, and these species are closely related to each other in phylogenetic trees (Supplementary Fig. S7; Jiang et al. 2023; Xu et al. 2025). Probably, the last common ancestor of these ciliates harbored *Methanocorpusculum*, and most of them retained their methanogenic endosymbionts. More anaerobic prostomatean species hosting methanogenic endosymbionts need to be investigated to test this hypothesis.

## Conclusion

In the present investigation, the identities of methanogenic endosymbionts of nine anaerobic ciliate populations were verified by autofluorescence, FISH, and amplicon sequences. For the first time, we reported methanogenic endosymbionts of anaerobic ciliates in the classes Muranotrichea and Prostomatea. Furthermore, we found that diverse anaerobic ciliates host methanogenic endosymbionts that were limited to a few genera in the orders Methanomicrobiales, Methanobacteriales, and Methanosarcinales. By adding these nine anaerobic ciliate populations, the evolution of associations between anaerobic ciliates and their methanogenic endosymbionts was re-analyzed. Distinct origins of endosymbiosis were revealed for the anaerobic classes Armophorea and Plagiopylea. Members of the class Armophorea probably harbored *Methanoregula* (order Methanomicrobiales) as methanogenic endosymbionts at the beginning of their evolution. By contrast, members of the class Plagiopylea probably harbored *Methanocorpusculum* (order Methanomicrobiales) as methanogenic endosymbionts at the beginning of their evolution. For both ciliate classes, independent endosymbiont replacement events occurred for methanogen-ciliate associations, which might have been caused by ecological transitions, species radiation of ciliate hosts, and/or vertical transmission bottlenecks of endosymbionts. Except for the class Armophorea, methanogenic endosymbionts were found in only one or two genera in other ciliate classes. Dense sampling of anaerobic ciliate genera hosting methanogenic endosymbionts across different classes is urgently needed to further improve our understanding about the evolution of their associations.

## Supplementary Information

Below is the link to the electronic supplementary material.

Supplementary file1 (PDF 4333 KB)

Supplementary file2 (XLSX 25 KB)

## Data Availability

16S rDNA V4 amplicons of methanogenic archaeal symbionts and free-living archaea in corresponding ciliate-free culture media (~ 400 bp) have been deposited in the NCBI Sequence Read Archive under BioProject PRJNA1134647. Ciliate 18S rDNA sequences and 16S rDNA sequences of methanogenic archaeal symbionts (~ 1 kb) have been submitted to GenBank (PQ005718–PQ005723, PQ005725–PQ005731, respectively).
