# Supplementary material for: Distinct evolutionary origins and mixed-mode transmissions of methanogenic endosymbionts are revealed in anaerobic ciliated protists
Source: Mar Life Sci Technol. 2025 May 13;7(4):700–16. doi: 10.1007/s42995-025-00295-9 (PMC12662959; doi:10.1007/s42995-025-00295-9)
Supplement: Supplementary file 1 — Supplementary file1 (PDF 4333 KB) [file 42995_2025_295_MOESM1_ESM.pdf]

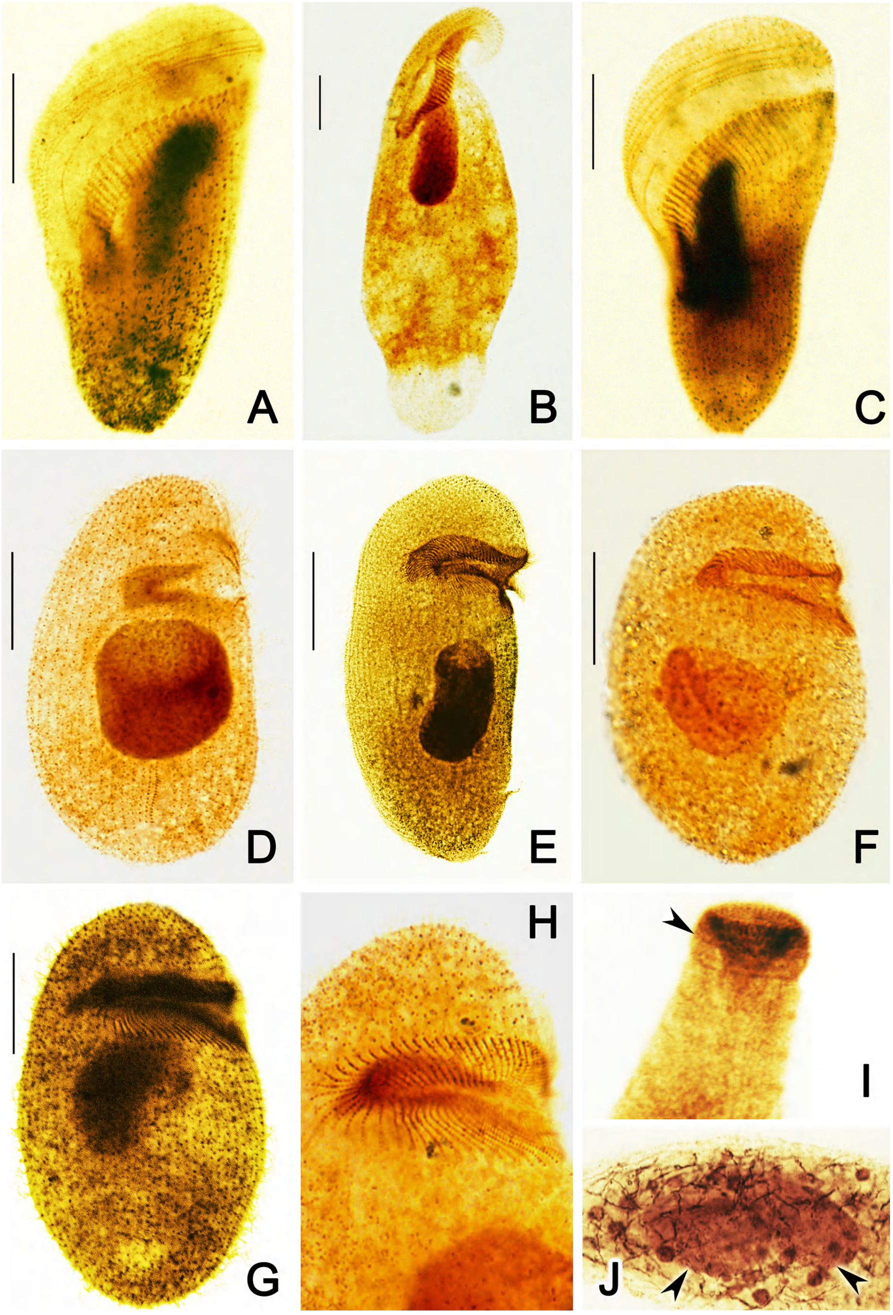

Fig. S1 Photomicrographs of *Metopus* cf. *contortus* (A), *M. laminarius* (B), *Metopus* sp. (C), *Plagiopyla* cf. *ramani* (D, H), *P. marina* (E), *P.* cf. *narasimhamurtii* pop. 2 (F), *P.* cf. *narasimhamurtii* pop. 1 (G), *Apolagynus* cf. *cucumis* (I, J) after protargol staining. Silver staining was not performed for *Thigmothrix strigosa*, and its 18S rDNA sequence is 100% identity to type species (GenBank accession number MT177192). A–J. Ventral and dorsal view of typical specimens, showing somatic kineties, macronucleus and oral region. H. Oral apparatus of *P.* cf. *ramani*. I, J. Oral apparatus and nuclear apparatus of *A.* cf. *cucumis*, showing perioral kineties (arrowhead in I) and macronucleus (arrowheads in J). Scale bar: 20 µm.

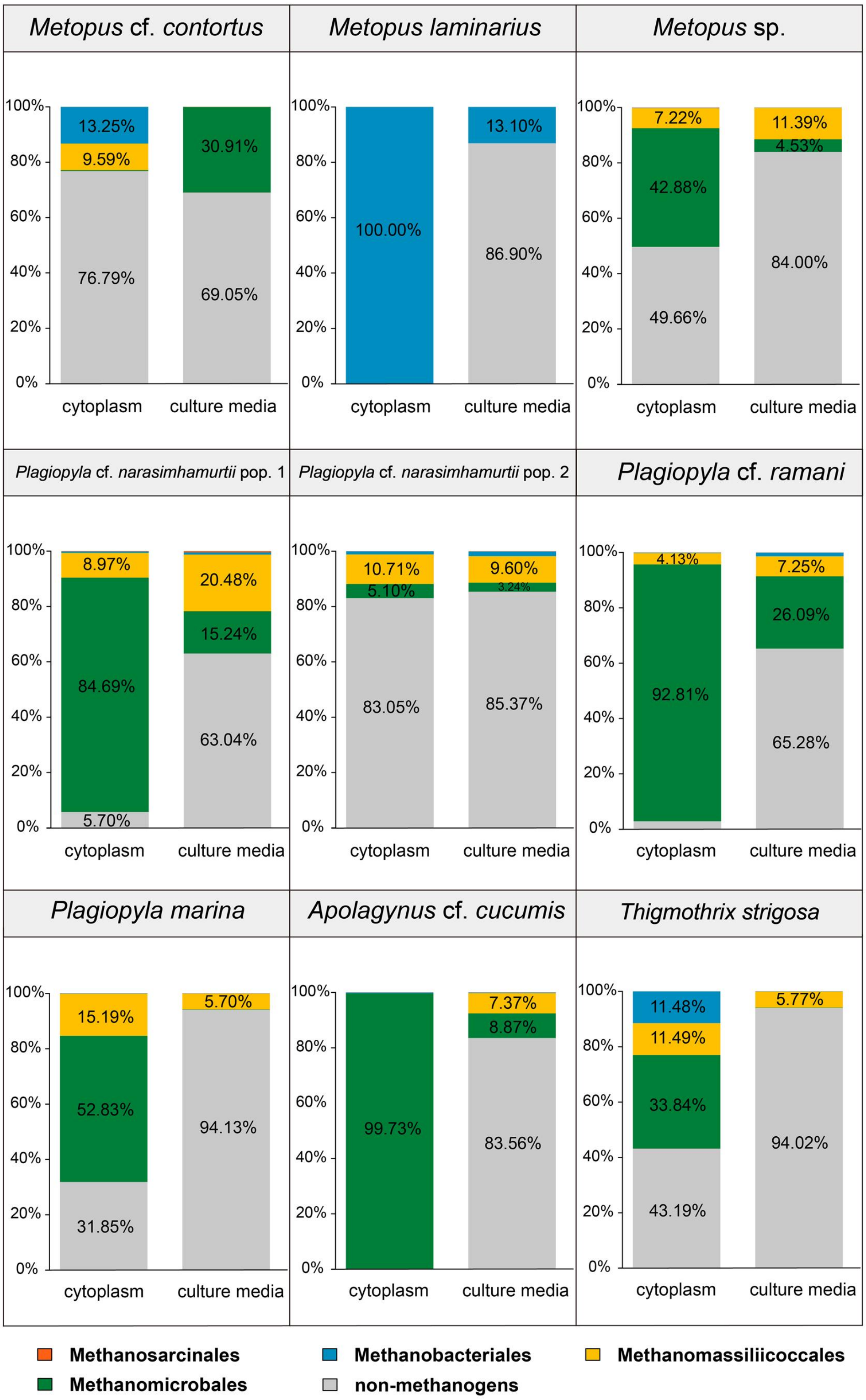

Fig. S2 Comparing abundance of 16S rDNA V4 amplicon reads between intracellular archaea in ciliate cells and free-living archaea in corresponding culture media. Ciliates from left to right and from top to bottom are *Metopus* cf. *contortus*, *M. laminarius*, *Metopus* sp., *Plagiopyla* cf. *narasimhamurtii* pop. 1, *P.* cf. *narasimhamurtii* pop. 2, *P.* cf. *ramani*, *P. marina*, *Apolagynus* cf. *cucumis*, *Thigmothrix strigosa*, respectively. The proportion of methanogenic orders with abundance less than 3.00% are not labeled due to limited space.

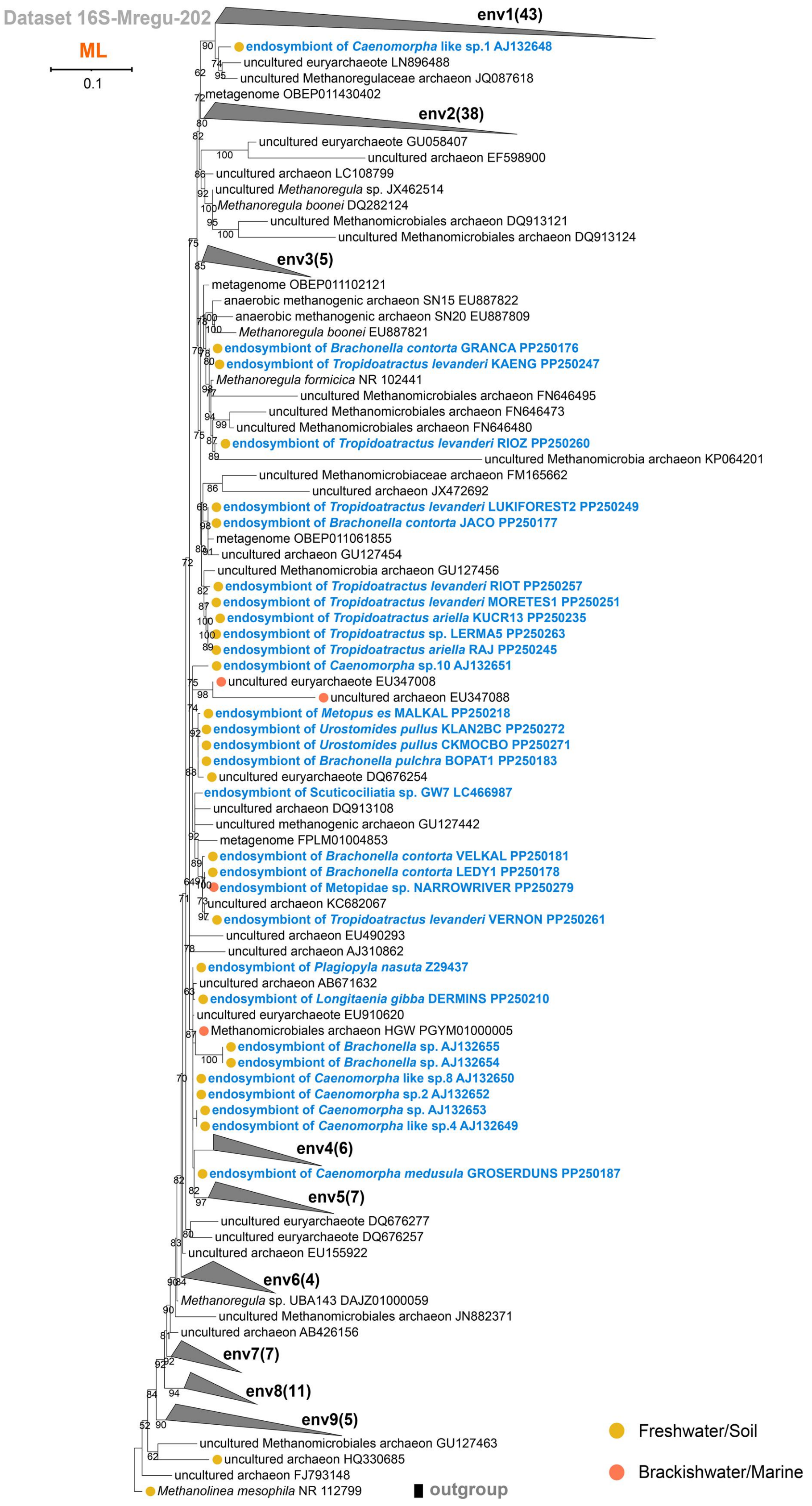

Fig. S3 Maximum likelihood (ML) tree of *Methanoregula* inferred from Dataset 16S-Mregu-202. The sequences of endosymbiotic methanogens in anaerobic ciliates published in GenBank are shown in blue bold font. Habitats of free-living methanogens or ciliate hosts are labeled with colored dots (yellow, freshwater/soil; red, marine/brackish water). The numbers at nodes represent bootstrap values of the ML. Support values below 50% are not shown.

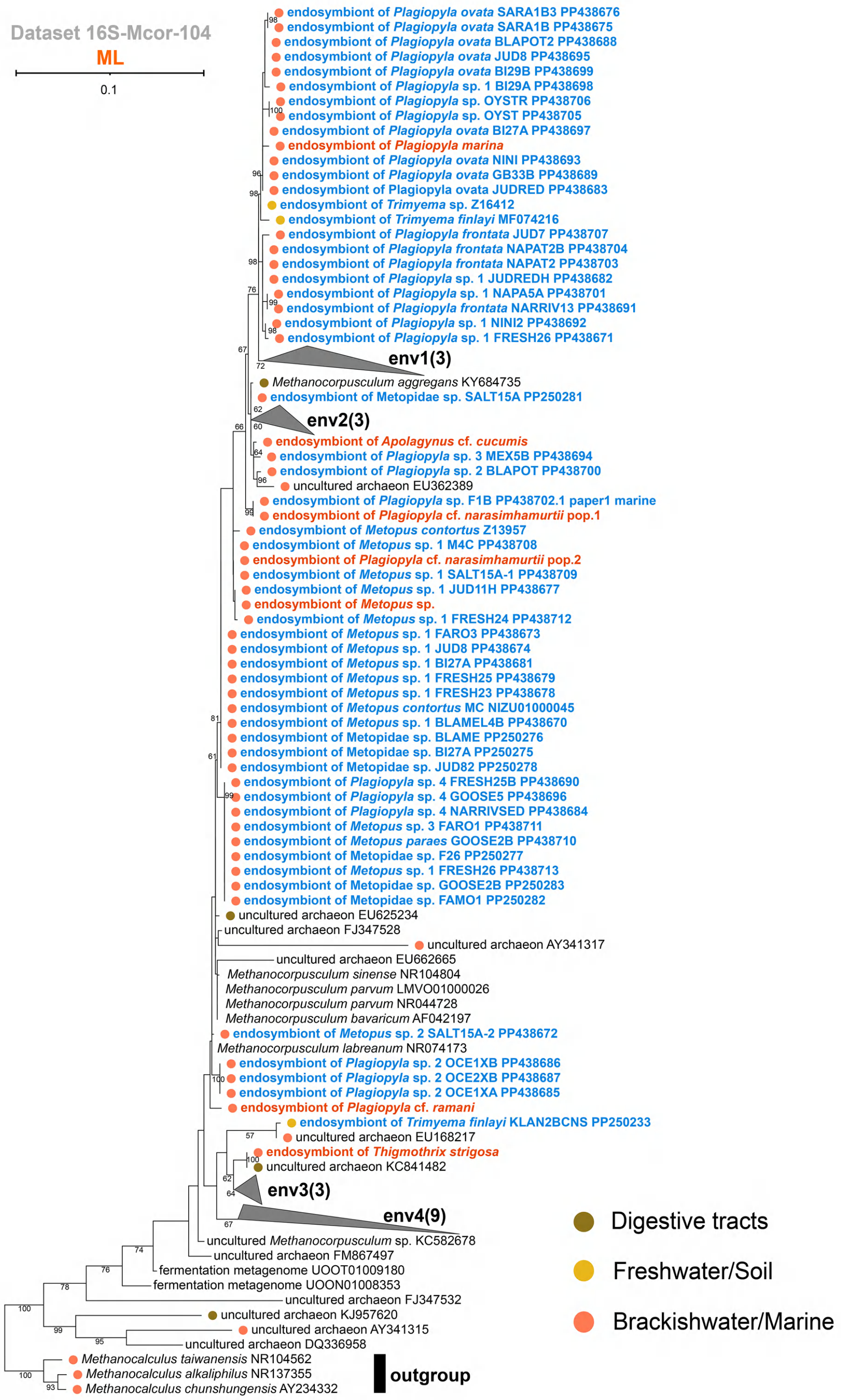

Fig. S4 Maximum likelihood (ML) tree of *Methanocorpusculum* inferred from Dataset 16S-Mcor-104. The sequences of endosymbiotic methanogens in anaerobic ciliates published in GenBank are shown in blue bold font, and the sequences newly obtained in this study are shown in red bold font. Habitats of free-living methanogens or ciliate hosts are labeled with colored dots (brown, digestive tract; orange, freshwater/soil; red, marine/brackish water). The numbers at nodes represent bootstrap values of the ML tree. Support values below 50% are not shown.

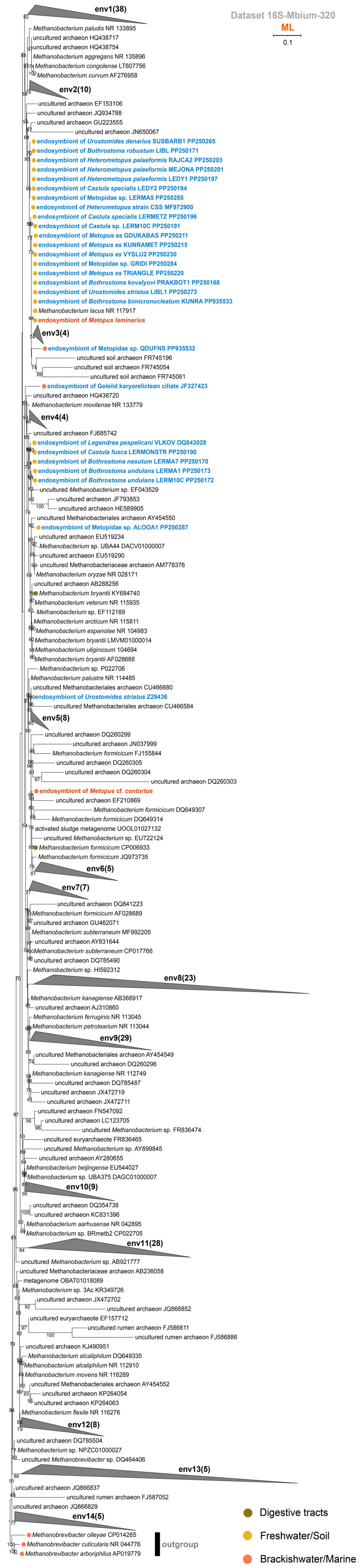

Fig. S5 Maximum likelihood (ML) tree of *Methanobacterium* inferred from Dataset 16S-Mbium-320. The sequences of endosymbiotic methanogens in anaerobic ciliates published in GenBank are shown in blue bold font, and the sequences newly obtained in this study are shown in red bold font. Habitats of free-living methanogens or ciliate hosts are labeled with colored dots (brown, digestive tract; orange, freshwater/soil; red, marine/brackish water). The numbers at nodes represent bootstrap values of the ML tree. Support values below 50% are not shown.

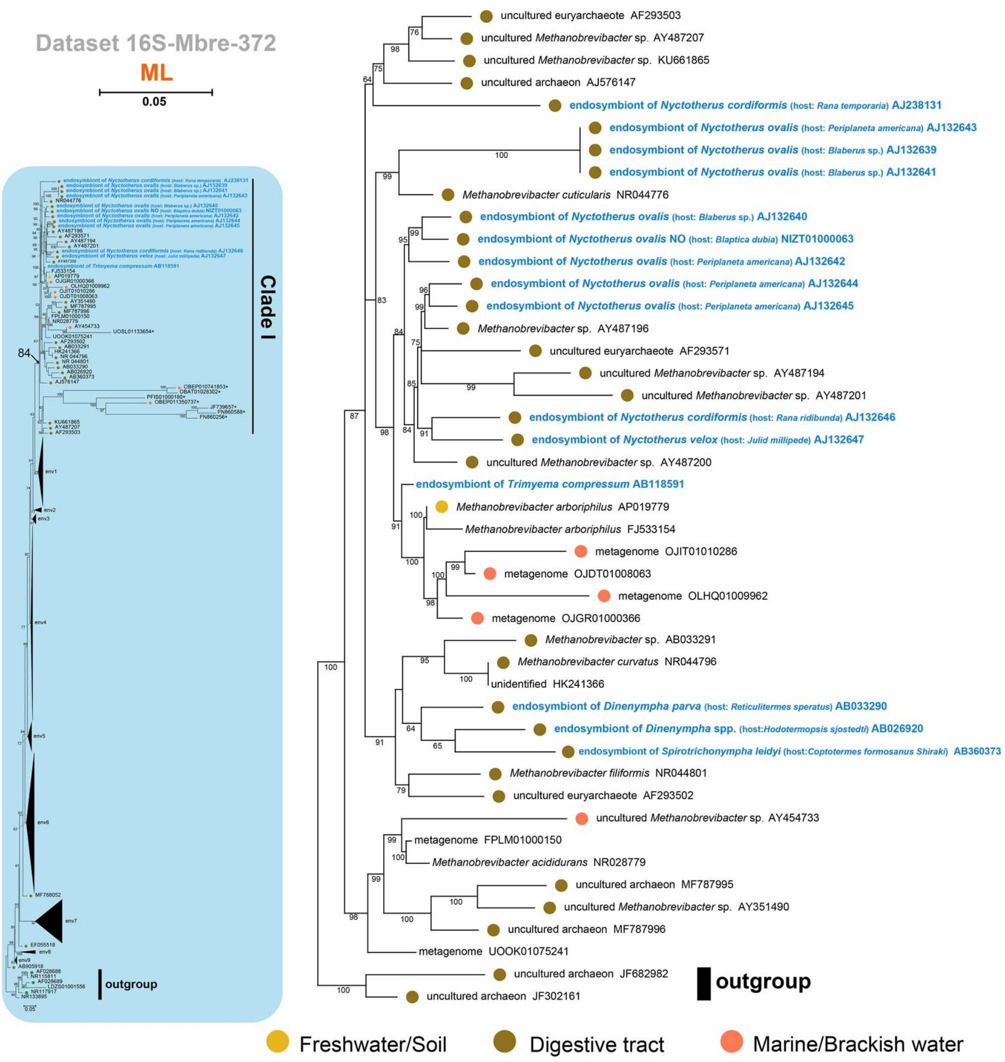

Fig. S6 Maximum likelihood (ML) tree of *Methanobrevibacter* inferred from Dataset 16S-Mbre-372 (left) and sequences other than those represented by "*" of Clade I (right). The sequences of endosymbiotic methanogens in anaerobic ciliates are shown in blue bold font. Habitats of free-living methanogens or ciliate hosts are labeled with colored dots (brown, digestive tract; orange, freshwater/soil; red, marine/brackish water). The numbers at nodes represent bootstrap values of the ML tree. Support values below 50% are not shown.

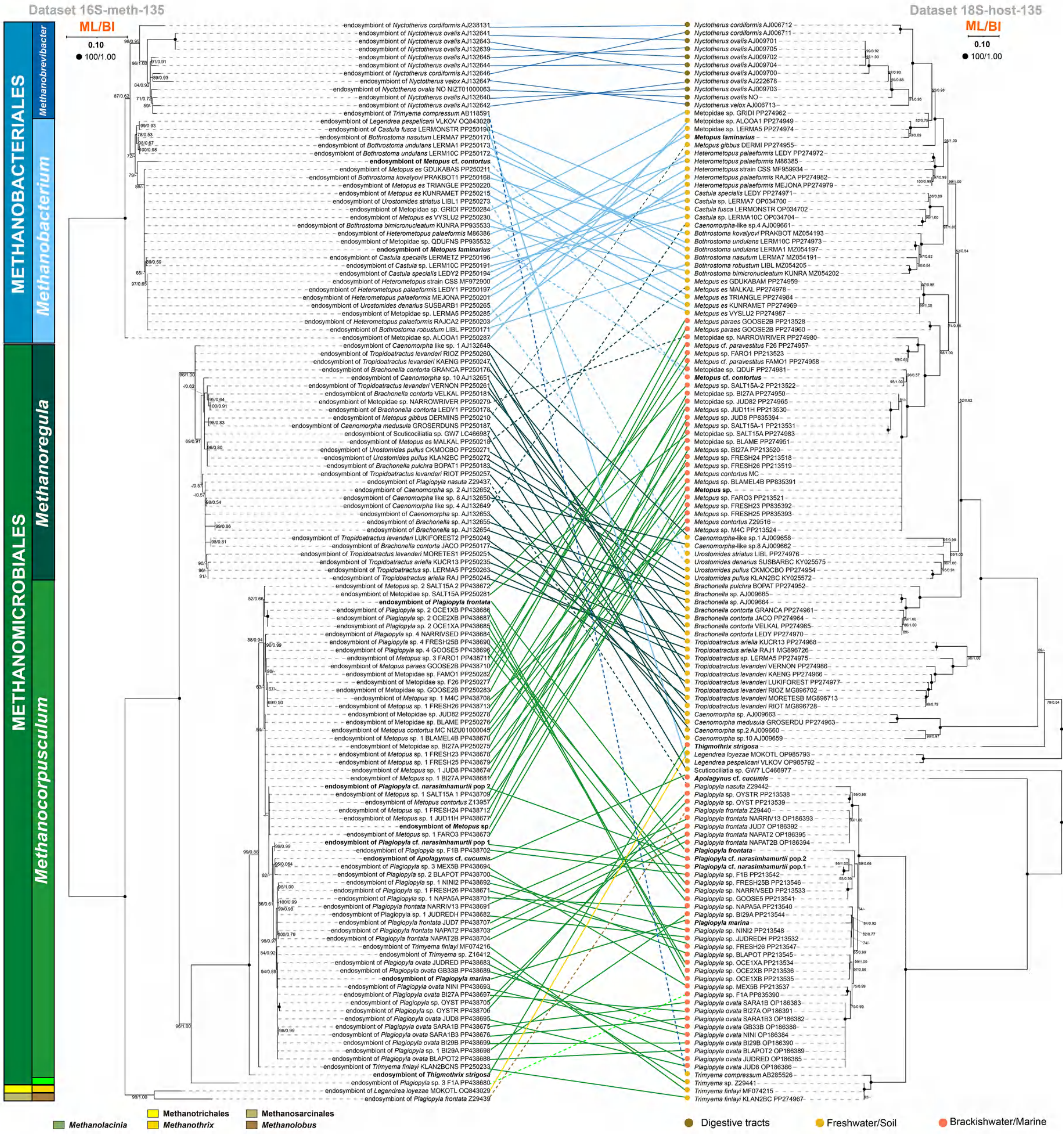

Fig. S7 Comparison of phylogenetic tree topologies between anaerobic ciliates of Dataset 18S-host-135 (right) and their endosymbiotic methanogens of Dataset 16S-meth-135 (left). The dash line represents a possible recent replacement event of endosymbiotic methanogens. Habitats of ciliate hosts are labeled with colored dots (brown, digestive tract; orange, freshwater/soil; red, marine/brackish water). Newly sequenced endosymbiotic methanogens and their ciliate hosts obtained in this study are shown in bold. The numbers at nodes represent bootstrap values of ML tree and posterior probabilities of BI tree, respectively. Support values equal to or below 50%/0.50 are not shown, and full support values are labeled with black dots.

Supplementary Table S1 Sampling information on anaerobic ciliate species studied in this investigation.

Class	Species	Sampling date	Sampling site	DO (mg/L)	Salinity (‰)
Armophorea	*Metopus* cf. *contortus*	2021/03/30	A sewage outlet at Dieshi Beach, Zhuhai, China (22°20′8″N, 113°35′31″E)	0.09	12
	Metopus sp.	2020/09/02	The intertidal zone of Zhanqiao Pier, Qingdao, China (36°06′N, 120°32′E)	--	14
	Metopus laminarius	2019/05/27	The Pearl River, Guangzhou, China (23°11′N, 113°38′E)	1.99	0
Plagiopylea	*Plagiopyla* cf. *narasimhamurtii* pop. 1	2021/03/31	Qi Ao Island, Zhuhai, China (22°25′34″N, 113°37′45″E)	0.30	12
	Plagiopyla cf. *narasimhamurtii* pop. 2	2019/02/21	Mangrove forest, Zhanjiang, China (21°12′N, 110°25′E)	0.23	18
	Plagiopyla cf. *ramani*	2020/11/17	Mangrove forest in Qi Ao Island, Zhuhai, China (22°25′38″N, 113°37′40″E)	0.36	11
	Plagiopyla marina	2020/09/02	The intertidal zone near Zhanqiao Pier, Qingdao, China (36°06′N, 120°32′E)	--	14
Prostomatea	*Apolagynus* cf. *cucumis*	2020/05/18	Jinsha Bay Beach, Zhanjiang, China (21°16′06″N, 110°23′08″E)	0.23	25
Muranotrichea	*Thigmothrix strigosa*	2020/09/02	The intertidal zone near Zhanqiao Pier, Qingdao, China (36°06′N, 120°32′E)	--	14

Note: DO, dissolved oxygen concentration

Supplementary Table S3 Probes used for fluorescence *in situ* hybridization (FISH).

**Probe**	**Sequence (5'-3')**	**Fluorophore**	**Specificity**	**Formamide (%)**	**Reference**
ARCH915	GTGCTCCCCCGCCAATTCCT	Cy3	Archaea	30	Raskin et al. (1994)
MG1200b	CRGATAATTCGGGGCATGCTG	6-FAM	Methanomicrobiales	30	Crocetti et al. (2006)
MB311	ACCTTGTCTCAGGTTCCATCTCC	Alexa Fluor 488	Methanobacteriales	20	Crocetti et al. (2006)
